# Ethanol metabolism and oxidative stress are required for unfolded protein response activation and steatosis in zebrafish with alcoholic liver disease

**DOI:** 10.1242/dmm.012195

**Published:** 2013-06-20

**Authors:** Orkhontuya Tsedensodnom, Ana M. Vacaru, Deanna L. Howarth, Chunyue Yin, Kirsten C. Sadler

**Affiliations:** 1 Division of Liver Diseases, Department of Medicine, Icahn School of Medicine at Mount Sinai, New York, NY 10029, USA; 2 Department of Developmental and Regenerative Biology, Icahn School of Medicine at Mount Sinai, New York, NY 10029, USA; 3 Department of Biochemistry and Biophysics, Programs in Developmental and Stem Cell Biology, Genetics and Human Genetics, Liver Center and Diabetes Center, Institute for Regeneration Medicine, University of California, San Francisco, CA 94158, USA; 4 Graduate School of Biomedical Sciences, Icahn School of Medicine at Mount Sinai, New York, NY 10029, USA

## Abstract

Secretory pathway dysfunction and lipid accumulation (steatosis) are the two most common responses of hepatocytes to ethanol exposure and are major factors in the pathophysiology of alcoholic liver disease (ALD). However, the mechanisms by which ethanol elicits these cellular responses are not fully understood. Recent data indicates that activation of the unfolded protein response (UPR) in response to secretory pathway dysfunction can cause steatosis. Here, we examined the relationship between alcohol metabolism, oxidative stress, secretory pathway stress and steatosis using zebrafish larvae. We found that ethanol was immediately internalized and metabolized by larvae, such that the internal ethanol concentration in 4-day-old larvae equilibrated to 160 mM after 1 hour of exposure to 350 mM ethanol, with an average ethanol metabolism rate of 56 μmol/larva/hour over 32 hours. Blocking alcohol dehydrogenase 1 (Adh1) and cytochrome P450 2E1 (Cyp2e1), the major enzymes that metabolize ethanol, prevented alcohol-induced steatosis and reduced induction of the UPR in the liver. Thus, we conclude that ethanol metabolism causes ALD in zebrafish. Oxidative stress generated by Cyp2e1-mediated ethanol metabolism is proposed to be a major culprit in ALD pathology. We found that production of reactive oxygen species (ROS) increased in larvae exposed to ethanol, whereas inhibition of the zebrafish CYP2E1 homolog or administration of antioxidants reduced ROS levels. Importantly, these treatments also blocked ethanol-induced steatosis and reduced UPR activation, whereas hydrogen peroxide (H_2_O_2_) acted as a pro-oxidant that synergized with low doses of ethanol to induce the UPR. Collectively, these data demonstrate that ethanol metabolism and oxidative stress are conserved mechanisms required for the development of steatosis and hepatic dysfunction in ALD, and that these processes contribute to ethanol-induced UPR activation and secretory pathway stress in hepatocytes.

## INTRODUCTION

Alcoholic liver disease (ALD) is a leading cause of liver-related deaths in the United States (Paula et al., 2010). Both chronic and binge drinking cause mitochondrial dysfunction (Mantena et al., 2008), secretory pathway stress (Ji, 2012) and lipid accumulation in hepatocytes (i.e. steatosis) (Rubin and Lieber, 1968). These cellular responses are largely attributed to the toxic byproducts of ethanol metabolism by hepatocytes and are central to ALD pathophysiology. An effective ALD treatment must combat these profound cellular defects; however, the mechanisms generating organelle stress and steatosis must first be uncovered. We propose that oxidative stress is one such mechanism.

Ethanol metabolism by alcohol dehydrogenase 1 (ADH1) generates highly reactive acetaldehyde that causes protein-acetaldehyde adducts. When ADH1 becomes saturated, ethanol is also metabolized by the endoplasmic reticulum (ER) membrane protein CYP2E1 (cytochrome P450, family 2, subfamily E, polypeptide 1) (Lieber, 1997), which produces both acetaldehyde and reactive oxygen species (ROS) such as 1-hydroxyethyl radical, superoxide anion and, most importantly, hydrogen peroxide (H_2_O_2_) (Wu and Cederbaum, 2009). An increased level of ROS results in lipid peroxidation, DNA and protein adduct formation, the depletion of glutathione stores, and a change in the cellular redox balance to favor oxidation. Thus, ethanol metabolism by CYP2E1 and oxidative stress are central to ALD pathology (Wu and Cederbaum, 2009).

Protein secretion by hepatocytes is severely impaired by ethanol (Baraona and Lieber, 1982; Howarth et al., 2012), and the subsequent serum protein deficiency causes clotting disorders, edema and impaired iron delivery (Beier et al., 2011), which underlie much of the morbidity and mortality in alcoholics. Activation of the unfolded protein response (UPR) is the cellular reaction to stress in the secretory pathway and, more specifically, to an accumulation of unfolded secretory cargo in the ER. The UPR is a complex pathway that serves to enhance and restore the protein-folding and secretory capacity of the ER (Walter and Ron, 2011). ER stress refers to sustained UPR activation due to unmitigated accumulation of unfolded proteins in the ER, reflecting organelle dysfunction that can result in steatosis and apoptosis (Imrie and Sadler, 2012). In contrast, moderate UPR activity is beneficial because it augments protein secretion and can serve as an adaptive mechanism to protect cells that are repeatedly stressed (Rutkowski and Kaufman, 2007). Therefore, it is important to understand how the UPR is regulated in both healthy and diseased cells.

**TRANSLATIONAL IMPACT**

**Clinical issue**

Alcoholic liver disease (ALD) is a leading cause of liver-related deaths in the United States. One of the primary effects of acute alcohol abuse is lipid accumulation in the liver (steatosis), a hallmark of ALD. Secretory pathway dysfunction is another common consequence of excessive alcohol consumption. The mechanisms underlying these defects are not completely understood, in part because many animal models of ALD do not develop the same hepatic defects following acute ethanol exposure. Moreover, although traditionally utilized rodent models of ALD are highly valuable, their use can be time-consuming and costly, emphasizing the need for new approaches. Zebrafish larvae are an attractive alternative vertebrate system to study the metabolic effects of alcohol owing to their large clutch size and rapid generation time, in conjunction with the simple culture system approach and the ability to manipulate the externally developing larvae. Here, zebrafish larvae were exploited to examine the relationship between ethanol metabolism, secretory pathway and oxidative stress, and hepatic steatosis.

**Results**

The authors expanded and refined a protocol that they previously developed to induce ALD in zebrafish larvae. In the present study, they use this model to address two important questions relevant to human ALD: (1) is ethanol metabolism required for the hepatic defects, and (2) is oxidative stress involved in secretory pathway defects and steatosis caused by ethanol? They report that alcohol-exposed larvae exhibit hepatic damage that is marked by changes in hepatic gene expression, hepatic stellate cell (HSC) activation, formation of reactive oxygen species, secretory pathway dysfunction, induction of the unfolded protein response, and steatosis. Blocking ethanol metabolism using 4-methylpyrazole and chlormethiazole, which inhibit the major metabolic enzymes required for ethanol metabolism in mammals, completely reversed alcohol-induced steatosis and reduced secretory pathway dysfunction in hepatocytes and HSC activation. Importantly, both blocking ethanol metabolism and reducing oxidative stress by administering antioxidants mitigated activation of the unfolded protein response and limited the formation of reactive oxygen species.

**Implications and future directions**

The thorough characterization of the hepatic response of zebrafish to ethanol confirms the mechanistic similarities between zebrafish and mammalian ethanol-metabolizing systems, validating the use of this model to investigate ALD pathophysiology. In line with this, several cellular phenotypes displayed by alcohol-exposed zebrafish recapitulate those observed in human ALD. Further studies are required to understand the complete enzymatic pathway involved in ethanol metabolism in zebrafish. The study also provides evidence that oxidative stress due to ethanol metabolism is central to the development of steatosis and to the defects in hepatocyte protein secretion, two fundamental cellular abnormalities induced by ethanol that contribute to the clinical sequelae of ALD. In the future, this model could be used to elucidate specific pathways that can be targeted therapeutically to treat this widespread disease.

Activation of the UPR by ethanol is conserved across vertebrates (Howarth et al., 2012; Ji, 2012; Ji and Kaplowitz, 2003; Malhi and Kaufman, 2011; Passeri et al., 2009) and reducing UPR activation has been shown to reduce alcoholic liver injury (Ji, 2012). Several studies point to a role for chronic UPR activation as a causative factor for steatosis in multiple etiologies of fatty liver disease (Cinaroglu et al., 2011; Henkel et al., 2009; Imrie and Sadler, 2012; Malhi and Kaufman, 2011; Puri et al., 2008; Rinella et al., 2011). Although the mechanistic link between the UPR induction and lipid metabolism is unknown, these studies suggest that UPR activation could be a widespread mechanism of steatosis. Despite the importance of secretory pathway dysfunction in ALD, the cause of UPR activation by ethanol is not known. In other systems, changes in the redox balance within the ER caused by excessive ROS can prevent proper disulfide bond formation, induce the accumulation of unfolded proteins and activate the UPR (Malhotra and Kaufman, 2007), and antioxidants can improve protein folding in the ER (Malhotra et al., 2008). These data suggest the hypothesis that ROS generated in hepatocytes by ethanol metabolism contributes to ER stress and steatosis in ALD. We test this hypothesis using zebrafish larvae exposed to alcohol.

Rodent models have been invaluable in identifying major pathophysiological mechanisms of ALD. However, these are expensive, time consuming and, as with most animal models of human disease, display distinct differences from the typical clinical presentation of alcoholics. We addressed the need for additional and complementary animal models of ALD by developing a protocol to induce ALD in larval zebrafish (Howarth et al., 2012; Howarth et al., 2013; Passeri et al., 2009; Yin et al., 2012). Among the most useful features of this system are the wide range of genetic and pharmacological tools available, the ability to carry out studies with sample sizes of hundreds of animals that far exceeds the scope of studies using rodents, a controlled environment with uniform nutrient delivery from the yolk, the small size and transparency of externally developing embryos and larvae, and the high genetic conservation between fish and humans (Howe et al., 2013). This applies to Adh1 and the full cadre of Cyp2 enzymes (Goldstone et al., 2010). Additionally, by 4 days post-fertilization (dpf), the liver is comprised of the major hepatic cell types found in mammals (Chu and Sadler, 2009; Field et al., 2003), and alcohol induces changes in both hepatocytes (Howarth et al., 2012; Passeri et al., 2009) and stellate cells (Yin et al., 2012). Moreover, fish are at the forefront of toxicology research and are thus particularly amenable to studies with waterborne xenobiotics, such as ethanol. These features allow us to assess multiple parameters across hundreds of animals in a single experiment, making zebrafish a powerful system to complement alcohol research in other vertebrates.

We provide a much needed, comprehensive evaluation of the response of zebrafish larvae to ethanol and establish that zebrafish metabolize ethanol by a mechanism similar to mammals. We then use this system to test the hypothesis that ethanol metabolism generates ROS, which contributes to ER stress and steatosis. We conclude that oxidative stress is a conserved aspect of ALD pathophysiology and that secretory pathway stress in ALD is, in part, due to oxidative stress.

## RESULTS

### Morphological abnormalities, ER stress and steatosis occur during acute and prolonged exposure to ethanol in zebrafish larvae

**Fig. 1.**
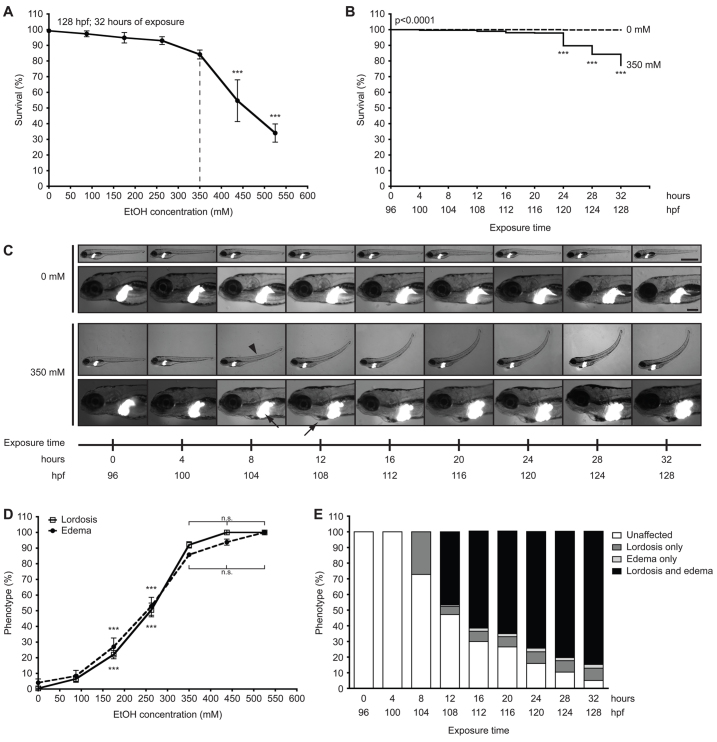
**Concentrations of ethanol exceeding 350 mM reduce survival and induce multisystemic morphological abnormalities in 4 dpf larvae.** (A) Larvae at 96 hpf were exposed to 0 mM, 87.5 mM (0.5%), 175 mM (1.0%), 262.5 mM (1.5%), 350 mM (2.0%), 437.5 mM (2.5%) and 525 mM (3.0%) ethanol and scored for viability at 128 hpf; mean ± s.e.m. *n*=6 clutches, *n*=125 larvae per treatment; ***P<0.001 versus 0 mM. The dashed line indicates the optimal concentration. (B) Kaplan-Meier survival curve of larvae exposed to 0 mM or 350 mM ethanol for 32 hours and scored for survival at every 4 hours; *n*=13 clutches, *n*=562 larvae per cohort. The *P*-value is indicated as determined by log-rank test; ***P<0.001 versus 96 hpf in 350 mM curve. (C) Images of one *Tg(fabp10:dsRed)* larva during exposure to 0 mM ethanol and another during exposure to 350 mM ethanol from 96 to 128 hpf. Arrowhead indicates lordosis at 104 hpf; arrows indicate hepatomegaly at 104 hpf and pericardial edema at 108 hpf. Scale bars: 1 mm in the upper panels and 0.2 mm in the lower panels. (D) Lordosis and edema were scored in larvae that survived 32-hour exposure to ethanol at concentrations of 0–525 mM; mean ± s.e.m. *n*=6 clutches, *n*=100 larvae. Except for the 87.5 mM ethanol group, all concentration points on both curves are significantly different (***P<0.001) compared with 0 mM. There is no significant (n.s.) difference in the percent of lordosis and edema in larvae treated with 350 mM ethanol or greater. (E) Morphological changes during 32 hours of exposure to 350 mM ethanol were averaged from ten clutches (*n*=442 per group). The percent of unaffected larvae was significantly reduced at all time points starting at 8 hours of exposure; the percent of larvae with lordosis alone was significantly increased from 8 hours of exposure; and the percent of larvae with both lordosis and edema was significantly increased at 12 hours of exposure and later; *P*<0.001. Untreated larvae (*n*=442) scored in parallel did not display any of these phenotypes at any time points (not shown). All statistical significance, except where indicated, was calculated by one-way ANOVA and Tukey’s post-hoc test.

Fully defining the mechanisms contributing to ethanol-induced toxicity and ALD in zebrafish necessitated a comprehensive analysis of the response of zebrafish larvae to ethanol. We chose to expose larvae to ethanol during a window after the liver is formed, at 96 hours post-fertilization (hpf), and before all yolk is utilized (5.5–6 dpf) to avoid the metabolic impact of fasting. We define acute exposure as 24 hours or less and 25–32 hours as prolonged exposure, which is distinct from the chronic exposure that occurs in alcoholics. Ethanol-induced mortality and morbidity were analyzed across six clutches with an average of 125 larvae per treatment through a dose response (Fig. 1A) and time course (Fig. 1B). Larvae exposed to a range of ethanol concentrations starting at 96 hpf and lasting up to 32 hours were scored for mortality (Fig. 1A). Concentrations below 350 mM did not induce significant mortality, whereas exposure to 437.5 mM or higher resulted in over 50% mortality, compared with 16% mortality caused by 350 mM (Fig. 1A). Most death caused by 350 mM ethanol occurred after 24 hours (Fig. 1B). This observation is consistent with previous conclusions that 350 mM is the maximal tolerable dose for prolonged exposure of larvae to ethanol (Passeri et al., 2009).

We previously reported that ethanol causes distinct morphological phenotypes, hepatomegaly and behavioral abnormalities in nearly all larvae after 32 hours of exposure to 350 mM ethanol (Howarth et al., 2011; Passeri et al., 2009). To determine when these morphological phenotypes occurred, we tracked individual 4 dpf *Tg(fabp10:dsRed)* larvae exposed to 350 mM ethanol over 32 hours (Fig. 1C) and verified these findings in larger cohorts (Fig. 1D,E). Hepatomegaly and lordosis were first observed at 8 hours, followed by edema in the pericardial region at 12 hours of exposure (Fig. 1C). The half maximal effective concentration of ethanol to induce lordosis and edema was 262.5 mM, and concentrations exceeding 350 mM did not increase the penetrance of these phenotypes (Fig. 1D), but did increase mortality (Fig. 1A). All phenotypes worsened over time, with the most significant changes occurring before 24 hours (Fig. 1C,E). By 32 hours of exposure to 350 mM ethanol, nearly all larvae displayed some phenotypic abnormality, albeit to varying degrees (supplementary material Fig. S1A,B).

**Fig. 2.**
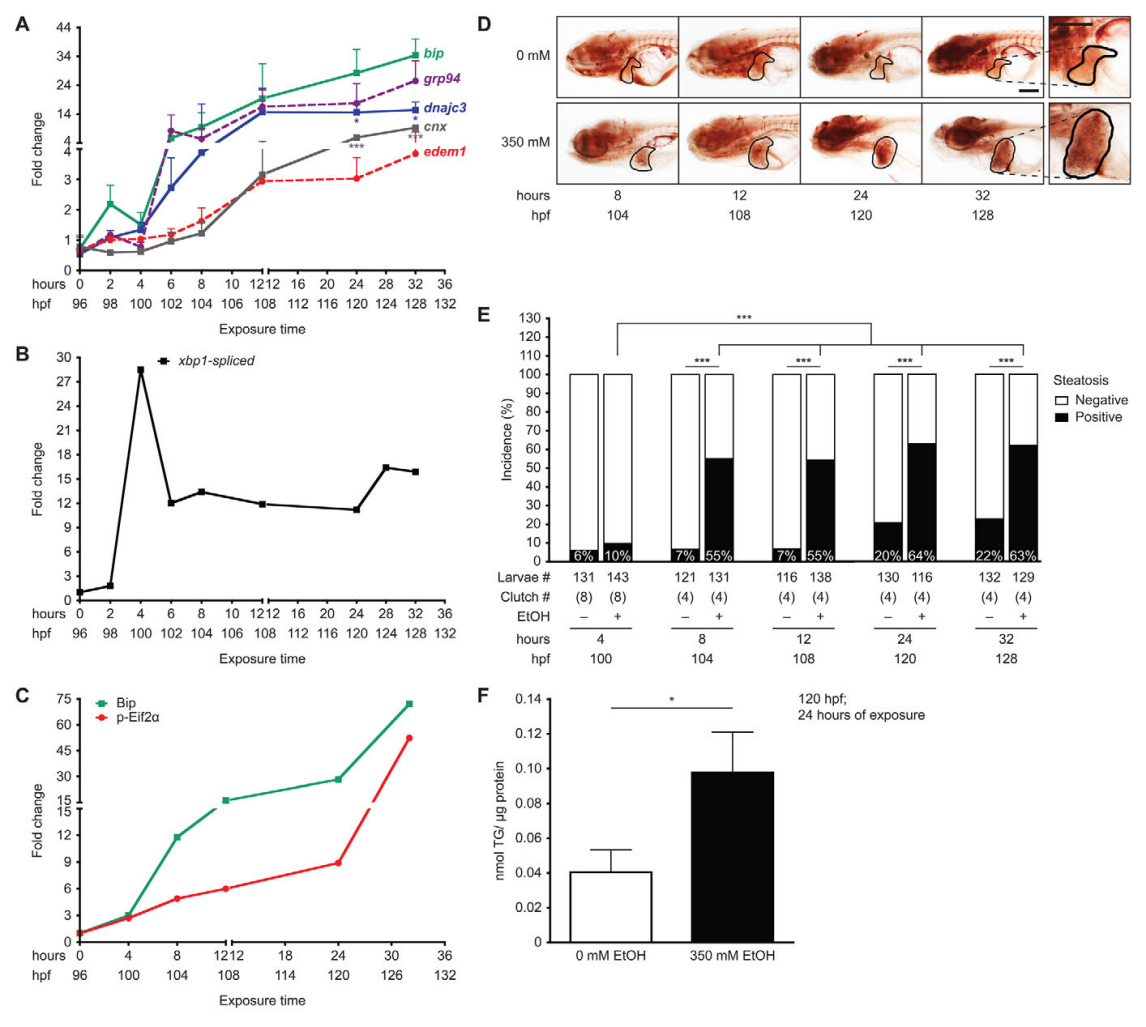
**Ethanol-induced UPR in the liver precedes steatosis.** (A) qPCR on cDNA prepared from pools of livers dissected from larvae exposed to 350 mM ethanol at 96–128 hpf. Fold changes were calculated by normalizing the comparative threshold (C_T_) values calculated as 2^−CT(^*^target)^*/2^−CT(^*^rpp0)^* to the ones obtained from 0 mM. ***P<0.001 and *P<0.05 by one-way ANOVA and Tukey’s post-hoc test. (B) Fold change in the percent of spliced *xbp1* from the total *xbp1* message present in liver cDNA from larvae exposed to 350 mM ethanol versus untreated controls, based on the PCR shown in supplementary material Fig. S2A. (C) Fold change in Bip protein levels and Eif2α phosphorylation normalized to β-actin was determined from the immunoblots in supplementary material Fig. S2B. (D) Representative images of whole-mount oil red O staining in larvae exposed to 0 or 350 mM ethanol at the indicated times. The livers are circled. The 32-hour image is enlarged to illustrate the lipid droplets used to score steatosis. Scale bar: 0.2 mm. (E) Average percent of steatosis across 4–8 clutches of larvae exposed to 0 (–) or 350 (+) mM ethanol. ***P<0.0001 by Fisher’s exact test. (F) Average triglyceride (TG) levels (nmol) in livers of larvae exposed to 0 or 350 mM ethanol for 24 hours were normalized to total protein (μg). Mean ± s.e.m. *n*=4 clutches, *P<0.05 by a Student’s *t*-test.

We previously demonstrated that alcohol causes steatosis (Howarth et al., 2011; Passeri et al., 2009), hepatic stellate cell (HSC) activation (Yin et al., 2012), and secretory pathway stress as marked by upregulation of the UPR (Howarth et al., 2012; Passeri et al., 2009). A time course analysis of UPR target gene expression in the liver during ethanol exposure revealed that mRNA levels of *bip*, a key chaperone and UPR target gene, as well as other ER resident chaperones (*grp94* and *dnajc3*) were induced in the liver by exposure to 350 mM ethanol after only 2 hours of treatment and that the level of induction was maximized after 6 hours of exposure (Fig. 2A). Other UPR targets [*edem1* and *calnexin* (*cnx*)] were significantly increased after 12 hours of treatment. The expression of most genes had a moderate decline between 2 and 4 hours of exposure followed by a continued increase at later time points. The initial wave of *bip*, *grp94* and *dnajc3* induction correlated with a marked, but transient, increase in *xbp1* splicing at 4 hours of ethanol treatment (Fig. 2B; supplementary material Fig. S2A). Finally, Bip protein expression and Eif2α phosphorylation were induced by 4 hours and continued to increase over time, with the highest induction occurring during the prolonged exposure (Fig. 2C; supplementary material Fig. S2B). We speculate that the biphasic nature of target gene activation and *xbp1* splicing reflects the transition from an adaptive UPR (at 2–12 hours) to a stressed UPR (24 hours and beyond) (Imrie and Sadler, 2012).

We next evaluated the incidence of steatosis during a time course of ethanol exposure using whole-mount oil red O staining (Fig. 2D,E). Although it is not as sensitive as other approaches that utilize advanced imaging (Carten et al., 2011) and reveal steatosis in a higher percentage of larvae, oil red O staining provides a rapid and high-throughput means to detect the incidence of fatty liver in zebrafish larvae, enabling us to examine steatosis across a large population of fish (i.e. the data in Fig. 2E represents results from 1287 larvae). We found a non-significant increase in steatosis after 4 hours of treatment but, by 8 hours, steatosis incidence robustly and significantly increased to 55% (Fig. 2E). There were further moderate increases after 12, 24 and 32 hours of ethanol exposure, but none reaching statistical significance (Fig. 2E). We did not assess the steatosis incidence past 32 hours because larvae consumed their yolk by 5.5 dpf (not shown) and thus developed fasting-induced steatosis (Cinaroglu et al., 2011). Interestingly, the incidence of steatosis in control larvae increased from less than 7% at 4 dpf (i.e. 4–12 hours of exposure) to over 20% at 5 dpf (24–32 hours; Fig. 2E). We speculate that these results reflected those larvae that might have already consumed their yolk and were entering the fasting state earlier than their siblings. The higher incidence of steatosis after 24 hours of ethanol exposure was due, at least partially, to the accumulation of triglycerides, which were more than doubled in the liver of ethanol-treated larvae compared with controls (Fig. 2F). In summary, UPR induction occurs as early as after 2 hours of exposure, whereas steatosis incidence increases significantly after 8 hours of ethanol treatment. Given that UPR activation is sufficient to cause steatosis in zebrafish (Cinaroglu et al., 2011; Thakur et al., 2011), as in mammals (Lee et al., 2012; Rutkowski et al., 2008; Teske et al., 2011; Zhang et al., 2011), our data suggest that UPR induction might also contribute to steatosis in ALD.

### Zebrafish larvae metabolize ethanol

**Fig. 3.**
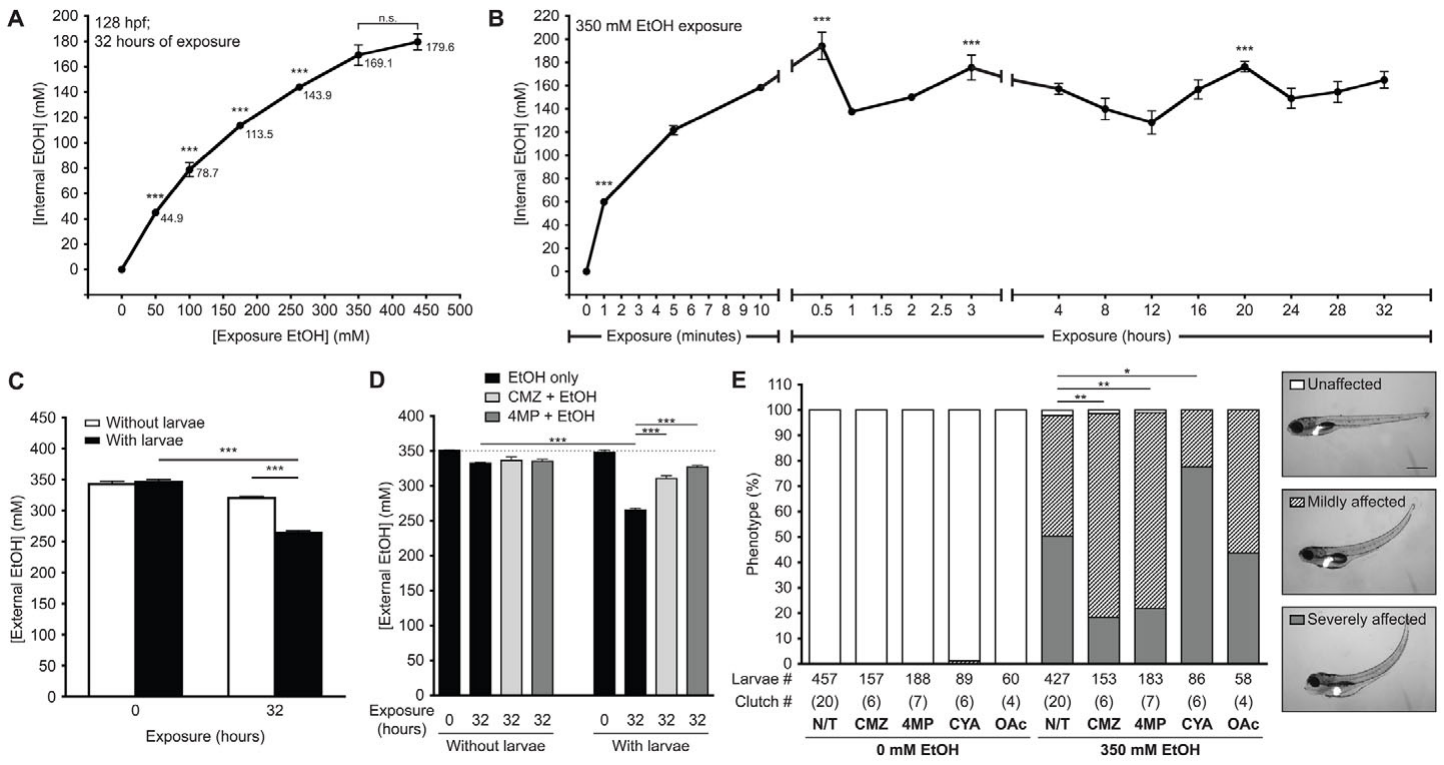
**Ethanol is rapidly internalized, utilized and metabolized in zebrafish larvae.** (A) Internal ethanol concentration was measured in homogenates of whole larvae treated with the indicated ethanol concentrations at 96–128 hpf. Values are in mM; mean ± s.e.m. *n*=6 clutches, *n*=260 larvae. All concentration points are statistically significant (***P<0.001) from the controls. Samples labeled as n.s. did not differ from one another. (B) Internal ethanol concentration determined from whole larvae treated with 350 mM ethanol for the indicated durations; mean ± s.e.m. *n*=6 clutches, *n*=200 larvae. All time points on the curve are significantly different (*P*<0.001) from *t*=0. ***P<0.001 versus *t*=32 hours. (C) Ethanol internalization and consumption was measured by calculating the external (water) ethanol concentration at *t*=0 and *t*=32 hours of larval exposure. To account for ethanol evaporation, concentration was also calculated from media that lacked larvae and was maintained in parallel. Values are the average of triplicate measurements of samples obtained from four clutches (mean ± s.e.m.); ***P<0.001. (D) The effects of CMZ and 4MP on ethanol utilization was measured in naïve larvae (black bars) or larvae pre-treated with 100 μM CMZ (light gray) or 1 mM 4MP (dark gray) at 94 hpf and then co-exposed to 350 mM ethanol from 96 to 128 hpf at a density of 1 larva/ml. Dashed line marks 350 mM ethanol. Values were calculated from triplicate measurements on three clutches (mean ± s.e.m.). ***P<0.001. (E) Untreated larvae (N/T) and larvae pre-treated with 100 μM CMZ, 1 mM 4MP, 3 mM CYA or 40 mM OAc at 94 hpf and then co-exposed with one of these drugs and 350 mM ethanol from 96 to 120 hpf were scored for mild or severe phenotypes, as shown in the representative images in the panels on the right. The severely affected phenotype (gray bars) was significantly reduced in larvae co-treated with CMZ or 4MP, but increased with CYA. *P<0.05 and **P<0.01. Statistics in all panels were calculated by one-way ANOVA and Tukey’s post-hoc test.

We next analyzed ethanol internalization as a function of dose (Fig. 3A) and time (Fig. 3B). The tissue concentration of larvae exposed to 50 and 100 mM ethanol for 32 hours was nearly equivalent to the external ethanol concentration (44.9 and 78.7 mM, respectively). However, exposure to higher ethanol concentrations resulted in a tissue concentration that was markedly lower than that in the culture medium: exposure to 350 and 437.5 mM ethanol resulted in average tissue concentrations of 169 and 179 mM, respectively (Fig. 3A), which were almost half the environmental concentration. A time course analysis of ethanol internalization showed that ethanol tissue concentration in 96 hpf larvae exposed to 350 mM ethanol approached 60 mM within 1 minute, peaked at 205 mM at 30 minutes and equilibrated to 140 mM by 1 hour, after which concentrations fluctuated between 150 and 175 mM (Fig. 3B). These findings suggest that zebrafish larvae efficiently utilize or excrete ethanol.

We measured ethanol utilization to determine the rate of ethanol metabolism. The ethanol concentration in the water before and after exposure of larvae to ethanol at a density of one larva per ml (Fig. 3C) was compared with plates without larvae to account for evaporation. After 32 hours, ethanol concentration was reduced from 350 to 265 mM in the plates containing larvae, significantly less than the decrease in concentration in plates without larvae (Fig. 3C). The average rate of ethanol consumption, hence ethanol metabolism, during this exposure protocol was calculated as 56 μmol/larva/hour.

### Ethanol metabolism via Adh1 and Cyp2 homologs is required for ethanol-induced toxicity

ALD in mammals is caused by toxic byproducts of alcohol metabolism where ethanol is oxidized by ADH1 and CYP2E1 to highly reactive acetaldehyde, which is further processed by aldehyde dehydrogenases (ALDH2 and ALDH1A1) to non-toxic acetate. Genes encoding three Adh enzymes (*adh5*, *adh8a* and *adh8b*) have been characterized in zebrafish (Dasmahapatra et al., 2001; Reimers et al., 2004), yet the *CYP2E1* ortholog has not been identified in any non-mammalian species. However, the lack of a direct *CYP2E1* ortholog does not mean that ethanol cannot be metabolized in other organisms; indeed, fish contain a rich diversity of Cyp2 genes and can metabolize a host of xenobiotics (Goldstone et al., 2010; Jang et al., 2012; Kaplan et al., 1991; Wall and Crivello, 1998). Zebrafish Cyp2y3 and Cyp2p6 are 43% identical to the human protein, and 42% identical to each other (supplementary material Fig. S3A), and represent the closest CYP2E1 homologs in zebrafish.

mRNA for *adh5*, *adh8a*, *adh8b*, *cyp2y3*, *cyp2p6* and *aldh2* were detected in the livers of 5 dpf zebrafish larvae (supplementary material Fig. S3B), and antibodies raised against human CYP2E1 and ADH1 detected single bands corresponding to proteins of the predicted size (57 kDa and 41 kDa, respectively; supplementary material Fig. S3C–E). Notably, the CYP2E1 immunoreactive protein was difficult to detect in the absence of ethanol (supplementary material Fig. S3C), but was stabilized by ethanol in a dose- and time-dependent manner (supplementary material Fig. S3C,E), as in mammals (Lieber, 1997). In contrast, expression of ADH1 immunoreactive protein did not change in response to ethanol (supplementary material Fig. S3D). Thus, the enzymes required for ethanol metabolism are present in the zebrafish liver.

To determine whether ethanol was metabolized by the ADH1 and CYP2 homologs in zebrafish, we took advantage of well-established pharmacological inhibitors of ADH1 and CYP2E1, 4-methylpyrazole (4MP) and chlormethiazole (CMZ). These inhibitors are particularly useful because they are thought to target multiple members of each family, and thus allowed us to investigate whether these classes of enzymes are required for ethanol metabolism and are responsible for the consequences of ethanol exposure in zebrafish.

CMZ is a specific ‘suicide’ inhibitor that destabilizes CYP2E1 (Lu and Cederbaum, 2006; Simi and Ingelman-Sundberg, 1999). Larvae tolerated exposures of up to 100 μM CMZ (supplementary material Fig. S4A), and co-treatment of 100 μM CMZ and ethanol reduced the levels of CYP2E1 immunoreactive protein in the livers (supplementary material Fig. S4B), indicating that CMZ destabilized the zebrafish CYP2 homologs, which we conclude are required for ethanol metabolism.

**Fig. 4.**
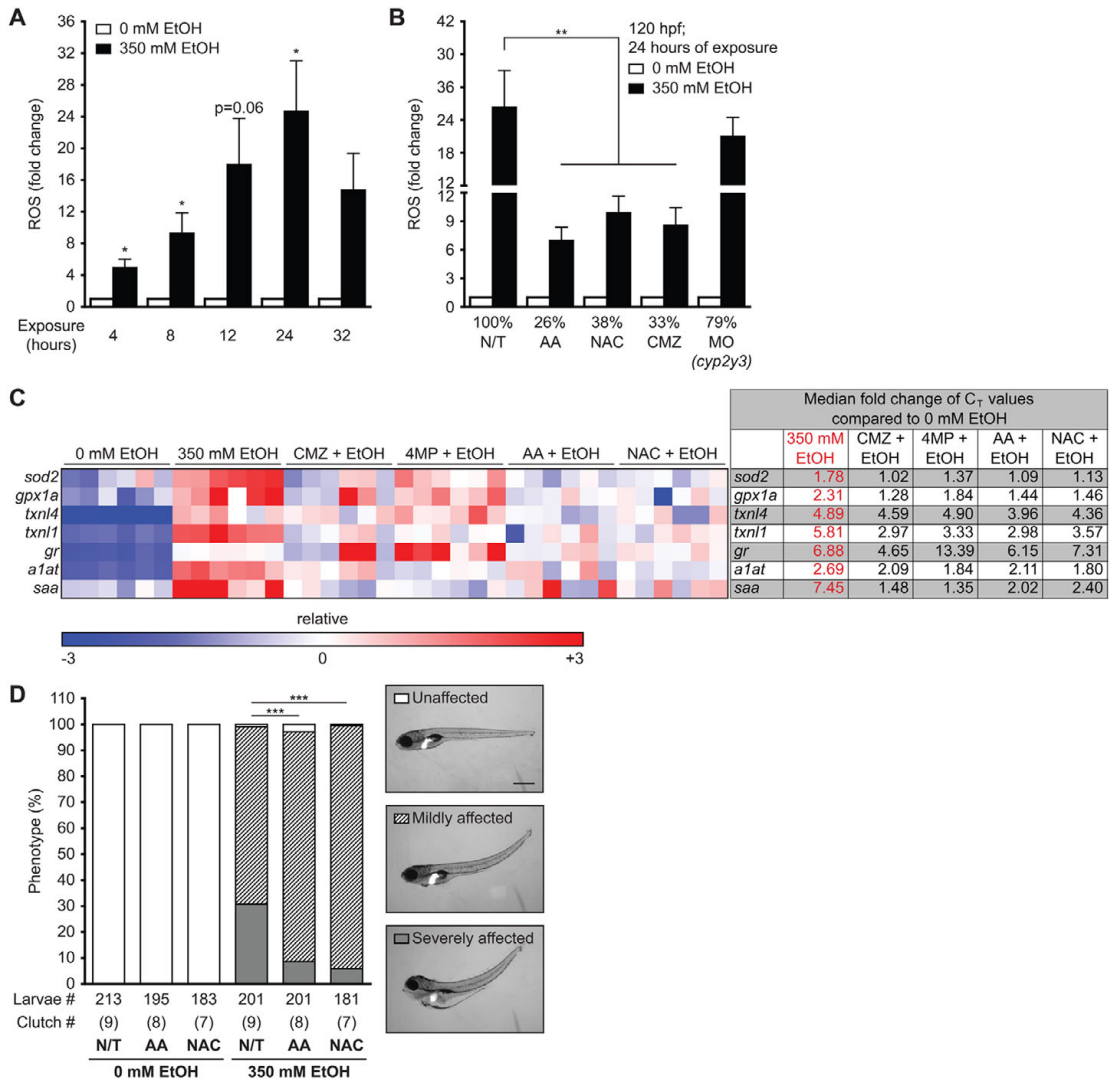
**Ethanol-induced ROS production and morphological abnormalities in zebrafish are rescued by antioxidants and inhibitors of ethanol metabolism.** (A) ROS production was measured by assaying CM-H_2_DCFDA fluorescence in the media during exposure to 350 mM ethanol. The arbitrary units of fluorescence measured in duplicate from larvae treated with 350 mM ethanol were normalized to corresponding untreated fish and the average fold changes of four clutches are shown. *P<0.05 as determined by a one-sample Student’s *t*-test. (B) Larvae were either pre-treated with 125 μM AA, 20 μM NAC or 100 μM CMZ at 94 hpf or injected with 4–6 nl of 0.1 mM *cyp2y3* morpholino at 0 hpf and then exposed to 350 mM ethanol at 96 hpf for 24 hours. **P<0.01 by one-way ANOVA and Tukey’s post-hoc test. (C) A heatmap of relative expression based on qPCR from cDNA isolated from the livers of larvae exposed to 350 mM ethanol alone or co-treated with CMZ, 4MP, AA or NAC for 24 hours. Each row is a gene and each column is a single clutch, and the color range (red – high, blue – low) was determined via the median method in GENE-E. CMZ, 4MP, AA and NAC treatments alone did not affect the expression of these genes when compared with untreated larvae (0 mM) and thus are not shown. The fold changes of the median C_T_ values of six clutches are shown to the right and individual C_T_ values are in supplementary material Table S2. The median was calculated for each row (gene) and subtracted from each data point. All six clutches are aligned according to the order of the lowest (blue) to the highest (red) expression of *sod2* in 350 mM ethanol treatment. (D) Representative images of unaffected, and mildly or severely affected larvae are shown on the right. The phenotypes were scored in an average of nine clutches (*n*=210 larvae per cohort). ***P<0.001 refers to severely affected fish (gray bars) and was calculated by one-way ANOVA and Tukey’s post-hoc test.

At low doses, 4MP is a selective inhibitor of ADH1 enzymes but, at higher doses, it acts as a stabilizing, competitive inhibitor of CYP2E1 (Wu et al., 1990). Concentrations of 4MP exceeding 1 mM stabilized the CYP2E1 immunoreactive protein in the liver (supplementary material Fig. S4D) and were well tolerated at concentrations up to 10 mM when administered alone. However, the maximal tolerable dose of 4MP was reduced to 3 mM when administered with ethanol (supplementary material Fig. S4C). We speculate that concentrations lower than 1 mM 4MP inhibit Adh1 but, at higher concentrations, it stabilizes CYP2 homologs. When ethanol is administered at concentrations capable of competing with 4MP for Cyp2 binding, it is preferentially metabolized through Cyp2 enzymes. Thus, by stabilizing CYP2 homologs, 4MP could be potentiating ethanol-induced injury. Regardless, 100 μM CMZ and 1 mM 4MP reduced ethanol utilization (Fig. 3D) from 67.4 μmol/larva/hour in controls to 26.1 or 8.2 μmol/larva/hour in CMZ- and 4MP-treated larvae, respectively (Fig. 3D).

**Fig. 6.**
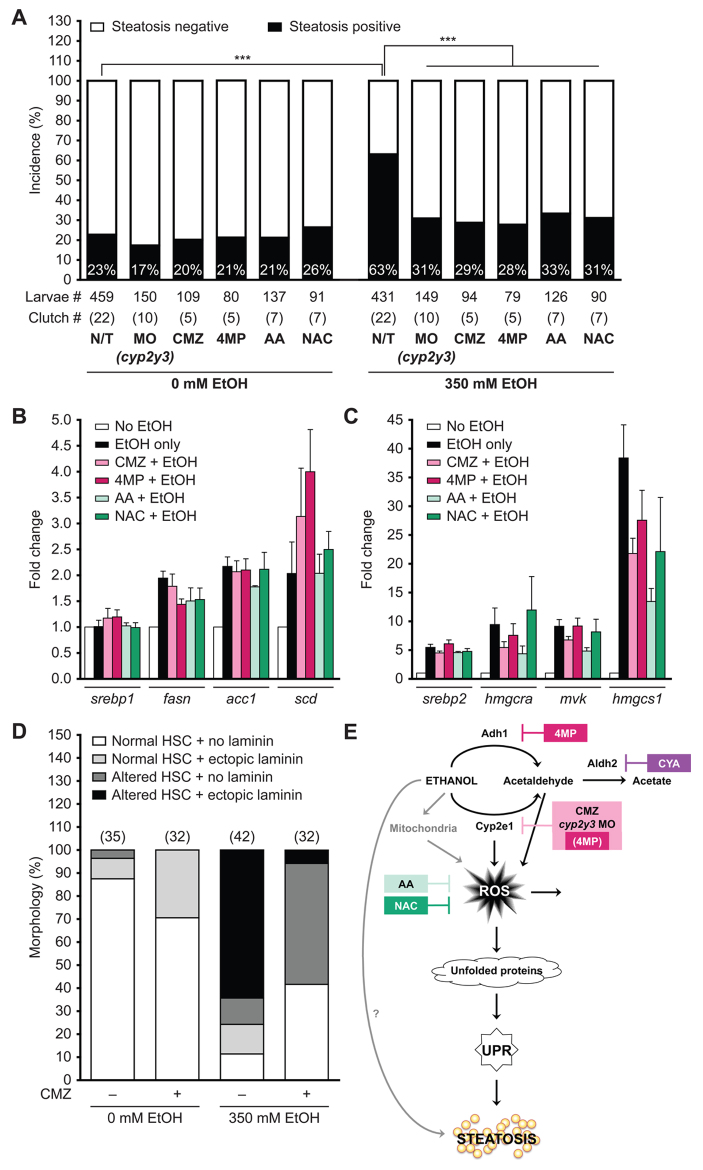
**Ethanol metabolism and ROS are required for steatosis and HSC activation.** (A) Quantification of whole-mount oil red O staining in 120 hpf larvae that were either untreated/uninjected (N/T), or injected with 4–6 nl of 0.1 mM *cyp2y3* morpholino solution at the one- to four-cell stage (0 hpf), or pre-treated with 100 μM CMZ, 1 mM 4MP, 125 μM AA, 20 μM NAC at 94 hpf and then exposed to 0 or 350 mM ethanol at 96 hpf for 24 hours. Note that, although each of these treatments did not affect the spontaneous steatosis levels, they significantly reversed the rate of ethanol-induced steatosis. ***P<0.0001 by Fisher’s exact test. (B,C) qPCR analysis of Srebp1 (B) and Srebp2 (C) target genes in cDNA isolated from the livers of untreated larvae or larvae exposed to 350 mM ethanol alone or co-treated with CMZ, 4MP, AA or NAC for 24 hours from six different clutches. Data are represented as mean fold changes to untreated samples with s.e.m. (D) Ethanol-induced HSC activation is partially rescued by CMZ. The percent of larvae with an altered HSC phenotype is an indication of activation, including laminin secretion (i.e. altered morphology + laminin). Over 70% of untreated control and CMZ-treated larvae have a normal HSC morphology with complex processes and no laminin deposition in the liver, whereas only 11% of larvae have some HSCs with a normal phenotype. Co-treatment of CMZ and ethanol increases the percent of larvae with normal HSCs to 42%. The number of larvae analyzed for each condition is indicated in parenthesis on top of each bar. (E) Working model illustrating that ethanol can generate ROS either through Cyp2-mediated metabolism (via acetaldehyde) or through altering mitochondrial metabolism and that high ROS levels directly cause protein damage. Together, these lead to unfolded protein accumulation in the ER, UPR induction and, by an as-yet-unknown mechanism, steatosis.

Ethanol-induced toxicity in zebrafish larvae is observed by distinct morphological changes (Fig. 1C–E) (Passeri et al., 2009). We hypothesized that these phenotypes result from ethanol metabolism to acetaldehyde by ADH1 and CYP2 homologs (see Fig. 6E) and by the generation of ROS (see below). 4MP and CMZ significantly reduced the ethanol-induced morphological changes (Fig. 3E). Aldh2 metabolizes acetaldehyde to acetate and the maximal tolerable concentration (3 mM; supplementary material Fig. S4E) of the Aldh2 inhibitor cyanamide (CYA) enhanced ethanol-induced phenotypes (Fig. 3E). In contrast, the maximal tolerable dose (40 mM; supplementary material Fig. S4F) of acetate (OAc) had no effect on larval phenotypes (Fig. 3E). These results suggest that acetaldehyde contributes, in part, to ethanol-induced phenotypes in zebrafish. Taken together, these data demonstrate that zebrafish metabolize ethanol by enzymes that are structurally similar to the mammalian system and thus are targeted by inhibitors of Adh1, Cyp2 and Aldh2. Whether these activities are attributed to single or multiple members of these enzyme families is an interesting topic for future studies.

### Ethanol metabolism generates ROS, oxidative stress and mediates ethanol toxicity

In mammals, ethanol metabolism by CYP2E1 increases ROS in hepatocytes (Caro and Cederbaum, 2004). Although the small size of the zebrafish larval liver prohibited obtaining accurate ROS measurements from isolated organs, we found that exposure to 350 mM ethanol increased ROS (H_2_O_2_, NO and ONOO^−^) to over four times the levels in untreated larvae by 4 hours of exposure and peaked to nearly 25-fold by 24 hours (Fig. 4A). ROS production was reduced by the antioxidants ascorbic acid (AA; 125 μM) and N-acetylcysteine (NAC; 20 μM), as well as by CMZ or by injecting a morpholino targeting *cyp2y3* (Fig. 4B), confirming that ethanol metabolism by CYP2 homologs generated ROS in zebrafish. Based on our finding that the phenotypic outcomes, ROS production and stabilization of the CYP2E1 immunoreactive protein, were all maximized by 24 hours of exposure to 350 mM ethanol and that mortality increased after this time point, the rest of our studies were carried out with 24 hours of exposure as an end point.

To replete the cellular antioxidant stores, genes encoding antioxidants are induced during oxidative stress. We previously reported that genes that respond to oxidative stress are induced in livers of zebrafish treated with ethanol (Howarth et al., 2013; Passeri et al., 2009). We expanded on it by using quantitative real-time PCR (qPCR) analysis of liver cDNA to investigate the expression of genes that mediate the antioxidant defense [*superoxide dismutase 2* (*sod2*), *glutathione peroxidase 1a* (*gpx1a*), *thioredoxin-like 1* and *4* (*txnl1*, *txnl4*) and *glutathione reductase* (*gr*)] as well as genes that encode the acute phase proteins that respond to oxidative stress and hepatic damage [*alpha one anti-trypsin* (*a1at*) and *serum amyloid* (*saa*); Fig. 4C]. As shown in the heatmap in Fig. 4C, all these genes were induced in the liver by ethanol, but their induction was reduced when co-treated with CMZ, 4MP, AA or NAC (Fig. 4C). None of the drugs affected the expression of these genes in the absence of ethanol (not shown). Additionally, AA and NAC co-treatments reduced the severity of ethanol-induced phenotypes (Fig. 4D). Therefore, we conclude that, as in mammals, ethanol metabolism generates ROS, which contributes to ethanol toxicity.

### Ethanol metabolism and ROS are required for ethanol-induced secretory pathway stress

**Fig. 5.**
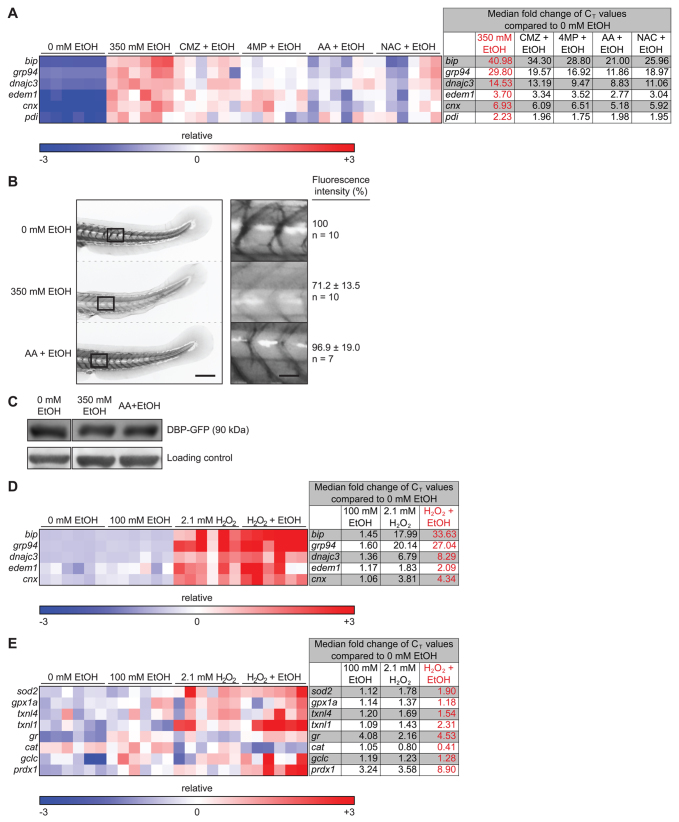
**Ethanol metabolism and ROS are required for secretory pathway stress.** (A) Heatmap of qPCR data from liver cDNA measuring the expression of genes involved in the UPR. The fold changes of the median C_T_ values are in supplementary material Table S3. All six clutches are aligned according to the order of the lowest to the highest expression of *bip* in 350 mM ethanol treatment. (B) Representative images of the tail of *Tg(l-fabp:Dbp-EGFP)* 120 hpf larvae treated with 0 or 350 mM ethanol or co-treated with AA and 350 mM ethanol for 24 hours. The rectangles in the left panel are magnified in the right panel. Scale bars: 0.2 mm for the left panel and 0.04 mm for the right panel. Note that the curved tail in untreated larvae is an artifact of fixation. (C) Immunoblots of transgenic *Tg(l-fabp:Dbp-EGFP)* larvae treated as in B using anti-GFP antibody and a non-specific band serving as a loading control. (D,E) Low concentration of ethanol and H_2_O_2_ synergize to induce UPR (D) and oxidative stress (E). Heatmaps of qPCR data from the livers of larvae exposed to individual treatments of 100 mM ethanol or 2.1 mM H_2_O_2_, or a co-treatment of the two, for 24 hours. The individual C_T_ values are in supplementary material Tables S4 and S5 for D and E, respectively. All six clutches are aligned according to the order of the lowest to the highest expression of *bip* (D) or *sod2* (E) in the co-treatment of 350 mM ethanol and 2.1 mM H_2_O_2_.

To determine whether ethanol metabolism and oxidative stress contributed to ethanol-induced secretory pathway stress, we first investigated the effects of 4MP, CMZ, AA and NAC on alcohol-induced UPR target genes (*bip*, *grp94*, *dnajc3*, *cnx*, *edem1*; Fig. 5A). As shown in the heatmap in Fig. 5A, 4MP, AA and NAC dramatically reduced the ability of ethanol to induce UPR target genes in the liver. Next, we monitored hepatocyte secretion using the transgenic line *Tg(l-fabp:Dbp-EGFP)*, in which a glycoprotein [vitamin D binding protein (Dbp)] fused to GFP (Dbp-EGFP) is expressed specifically in hepatocytes (Xie et al., 2010). Ethanol exposure reduced the levels of circulating fluorescent Dbp-EGFP (see Howarth et al., 2013) (Fig. 5B), but did not reduce the total Dbp-EGFP protein levels (Fig. 5C), suggesting that either secretion or correct folding to generate the fluorophore was impaired by ethanol. Circulating fluorescence levels were restored by co-administration of AA and ethanol (Fig. 5B).

Our data suggest that oxidative stress contributes to hepatic secretory pathway stress in ALD, which predicts that ROS should synergize with ethanol to induce the UPR. We identified 2.1 mM H_2_O_2_ and 100 mM ethanol as sub-threshold concentrations because each induced a moderate amount of ROS (supplementary material Fig. S5) but did not cause any morphological phenotypes or mortality when administered alone to 4 dpf larvae (Fig. 1A,D; supplementary material Fig. S5A). The combination of 2.1 mM H_2_O_2_ and 100 mM ethanol synergized to induce more than 50% mortality (supplementary material Fig. S5C) and to upregulate UPR target gene expression in the liver (Fig. 5D). Additionally, whereas 100 mM ethanol or 2.1 mM H_2_O_2_ produced a modest upregulation of *sod2*, *gpx1a*, *txnl1*, *txnl4*, *gr*, *glutamate-cysteine ligase catalytic subunit* (*gclc*) and *peroxiredoxin 1* (*prdx1*), their expression levels additively, if not synergistically, increased when these treatments were combined (Fig. 5E). Therefore, we conclude that oxidative stress is sufficient to cause secretory pathway dysfunction in hepatocytes and that ROS contributes to UPR induction in ALD.

### Ethanol metabolism and ROS contribute to steatosis and HSC activation in ALD

Steatosis and HSC activation are central aspects of ALD pathophysiology. Fig. 6A shows that ethanol-induced steatosis was significantly reduced by inhibition of ethanol metabolism, either by *cyp2y3* knockdown, or by treatments with CMZ or 4MP, and was also reduced by co-administration of AA or NAC (Fig. 6A), but not by the Aldh2 inhibitor CYA or by accumulation of acetate (OAc) (supplementary material Fig. S6E). Sterol regulatory element-binding protein 1 (Srebp1) and 2 (Srebp2) transcription factors contribute to alcohol-induced steatosis in zebrafish (Passeri et al., 2009) and mice (Hu et al., 2012; You et al., 2002). We found that CMZ, 4MP, NAC and AA reduced the ability of ethanol to induce some Srebp1 (Fig. 6B) and Srebp2 (Fig. 6C) target genes, albeit not to baseline.

Finally, HSC activation assessed in *Tg(hand2:EGFP)^pd24^* larvae (Yin et al., 2012) was increased in response to ethanol, as shown by changes in HSC morphology including an elongated cell body, loss of cellular processes and clustering of the HSCs (supplementary material Fig. S6C″), and increased laminin deposition (supplementary material Fig. S6C′). CMZ alone did not affect HSC morphology but did partially decrease the phenotypes characteristic of HSC activation (Fig. 6D). Thus, we conclude that ethanol metabolism by ADH1 and CYP2 homologs is required for fatty liver and HSC activation in zebrafish.

## DISCUSSION

We pioneered zebrafish larvae to study ALD (Howarth et al., 2012; Howarth et al., 2013; Passeri et al., 2009). Here, we use this system to examine the interplay between ROS generated from ethanol metabolism and major aspects of ALD pathology – steatosis and secretory pathway stress in hepatocytes. To address these and other important issues that arise with the development of a new animal model, we performed a detailed analysis of the response of zebrafish to ethanol. We confirmed and expanded on previous findings (Howarth et al., 2011; Passeri et al., 2009) that 350 mM ethanol is the maximal tolerable dose for acute and prolonged exposure of zebrafish larvae. Our data indicate that hepatic damage, measured by changes in hepatic gene expression, steatosis, UPR activation and secretory pathway dysfunction as well as HSC activation, occur during the acute phase of exposure, analogous to an alcohol binge. Prolonged exposure causes further deterioration of the liver. Because exposed larvae are immotile and cannot feed once yolk is depleted, the effects of chronic exposure were not addressed.

Our data demonstrate that zebrafish homologs of the mammalian ethanol metabolizing machinery – ADH1 and CYP2E1 – are expressed in the liver and function to metabolize ethanol in zebrafish. Although the small size of larval zebrafish means that they are not amenable to experiments directly measuring enzyme activity or ROS levels in isolated livers, by measuring ethanol utilization and ROS production in total larvae we found that that blocking ADH1 and CYP2 homologs in zebrafish significantly reduced ethanol metabolism, ROS levels, oxidative stress and every other aspect of ALD investigated. We used 4MP at a dose that we presumed to primarily target Adh1, although the improved efficacy of 4MP in most of our assays might indicate that some inhibition of CYP2 homologs occurred, suggesting that both enzymes were responsible for the effects of alcohol on zebrafish. We were unable to completely reverse the effects of ethanol by the inhibitors 4MP and CMZ, which could be attributed to issues of compound stability, toxicity or bioavailability.

Whereas Adh1 enzymes are relatively well conserved (Dasmahapatra et al., 2001; Reimers et al., 2004), the Cyp2 family expanded during fish evolution, perhaps as a consequence of selective pressure exerted by exposure to waterborne toxins. An antibody against human CYP2E1 detects a single species on immunoblots of zebrafish livers, which is stabilized in response to CYP2E1 substrates and is reduced in response to CMZ. However, many Cyp2 proteins have the same molecular weight and the anti-CYP2E1 antibody could recognize several isoforms. Therefore, the *cyp2y3* morpholino might have failed to reduce the CYP2E1 immunoreactive protein (not shown), although limited morpholino efficacy at late developmental stages could also account for this finding. Our focus in this study was to investigate whether the basis for ALD in zebrafish requires the same basic mechanisms as reported in mammals, and then to use this model to identify the mechanism of steatosis, HSC activation and secretory pathway stress. To this end, we demonstrate that zebrafish homologs of CYP2E1, including Cyp2y3, are required for ALD in zebrafish. Future studies will be required to decipher the exact Cyp2 and Adh1 family members involved in ethanol metabolism in zebrafish.

Although we found several similarities between zebrafish and mammals, which will further advance the use of zebrafish to study ALD, there are some noted differences. First, the internal concentration of ethanol in zebrafish tissues exceeds the blood alcohol concentration achievable in humans, but it is important to note that larval ethanol tissue concentrations and blood alcohol levels cannot be compared directly because the nature of the samples are different. However, it is likely that the concentration of alcohol tolerated by zebrafish exceeds that of mammals. This could be attributed to the fact that zebrafish swallow to maintain oxygen perfusion of their gills, and we presume that the gastrointestinal tract, gills and even diffusion through the skin might all provide a rapid means for ethanol entry. In contrast, mammals internalize ethanol by oral ingestion, which is paced. Additionally, because fish coexist with waterborne toxins, they evolved sophisticated mechanisms of detoxification (Di Giulio and Hinton, 2008; Kaplan et al., 2001; Kaplan et al., 1991; Nebert et al., 1989; Nelson, 1999; Wall and Crivello, 1998). Thus, zebrafish are predicted to be tolerant of higher levels of ethanol than are their mammalian counterparts.

Based on our finding that antioxidants block steatosis and reduce UPR induction, and that low doses of H_2_O_2_ and ethanol synergize to induce the UPR, we conclude that ethanol-induced oxidative stress causes dysfunction in the hepatocyte secretory pathway. ROS contributes to ER stress by altering the redox balance, which is important for oxidative protein folding in the ER (Fig. 6E) (Malhotra and Kaufman, 2007). In ALD, ROS is generated by ethanol metabolism by ER-localized Cyp2 enzymes, but might also come from interaction between the ER and the mitochondria, whereby a disrupted electron transport chain generates ROS, which could be directly transferred to the ER by the channels that connect these organelles (Csordás et al., 2006). Regardless of the source, we propose that ROS is a central mechanism that impairs hepatocyte secretory pathway function in ALD.

Although we do not directly demonstrate a causative relationship between UPR activation and steatosis, a number of studies in fish and mammals clearly show a direct link between ER stress and fatty liver. For instance, we and others have demonstrated that ER stress induced by blocking protein glycosylation with tunicamycin is sufficient to cause steatosis (Cinaroglu et al., 2011; Henkel et al., 2009; Imrie and Sadler, 2012; Malhi and Kaufman, 2011; Puri et al., 2008; Rinella et al., 2011). However, whether the same constellation of UPR targets and effectors that become activated in this robust ER stress are also active in response to ethanol is not clear. Evaluating how different UPRs contribute to fatty liver and hepatic injury in ALD and in other etiologies of this disease is of great interest. Collectively, our findings indicate that zebrafish metabolize alcohol in a similar fashion to humans, that ROS is central to ALD in this system, and that these processes contribute to secretory pathway stress and steatosis in ALD.

## MATERIALS AND METHODS

### Zebrafish maintenance, treatment and scoring

Adult wild-type zebrafish (Tab14), and transgenic zebrafish lines *Tg(fabp10:dsRed)* (Korzh et al., 2008), *Tg(l-fabp:Dbp-EGFP)* (Xie et al., 2010) (a kind gift from Drs Torres-Vázquez and Anand-Apte) and *Tg(hand2:EGFP)^pd24^* (Yin et al., 2012; Yin et al., 2010) were maintained on a 14:10 hour light:dark cycle at 28°C. Fertilized embryos were collected following natural spawning, and raised at 28°C according to standard procedures. The Institutional Animal Care and Use Committees of Icahn School of Medicine at Mount Sinai and University of California San Francisco approved all zebrafish protocols.

Ethanol (Pharmco-AAPER; Brookfield, CT) and H_2_O_2_ was administered directly to the larval incubation water at 96 hpf for up to 32 hours. Treatments with 100 μM CMZ, 1 mM 4MP, 3 mM CYA, 40 mM sodium acetate (OAc), 125 μM AA or 20 μM NAC (all from Sigma-Aldrich; St Louis, MO) began at 94 hpf and then were co-treated with 350 mM ethanol starting at 96 hpf. In other experiments, 2.1 mM (0.005%) H_2_O_2_ (VWR; Radnor, PA) was used as a co-treatment with 100 mM ethanol starting at 96 hpf and lasting for 24 hours. Larvae were treated at a constant density of 1 larva/ml. Plates were sealed with parafilm to minimize evaporation incubated at 28°C. Livers were dissected from 3–20 larvae and pooled for protein, RNA extraction or triglyceride determination.

Lordosis was scored as curvature of the tail ranging from 10–45 degrees (mild) to >45 degrees (severe). Edema was scored as moderate enlargement of the pericardial sac (mild) to anasarca (severe). Liver size was scored as normal if the left liver lobe was thin and crescent shaped; mild hepatomegaly was denoted by an enlarged but crescent-shaped liver; and severe hepatomegaly was represented by a large, circular liver.

A translation-blocking morpholino against *cyp2y3* (5′-CTCCATTCCTCTTACCGATCAGTTC-3′; the sequence that binds the initiating methionine is underlined) was purchased from Gene Tools, LLC (Philomath, OR). On average, 4–6 nl of a 0.1 mM stock concentration was injected into over 50 embryos prior to the four-cell stage.

### Whole-mount oil red O staining

Larvae were fixed in 4% paraformaldehyde in 1× PBS overnight at 4°C and stained with 0.5% oil red O in propylene glycol and scored for steatosis as described (Howarth et al., 2013).

### Triglyceride determination

Livers from untreated larvae and those treated with 350 mM ethanol (*n*≈20 each) were dissected and lysed in 0.5% Triton X-100 and heated at 80°C for 5 minutes. Triglycerides were measured from the liver lysate using the Infinity^TM^ Triglyceride Liquid Stable Reagent (Thermo Fisher Scientific, Waltham, MA) following the manufacturer’s instructions, and were normalized to the total protein concentration as determined by Bradford Assay (Bio-Rad, Hercules, CA).

### RNA extraction and PCR

Total RNA was isolated from pools of 3–15 dissected livers using TRIzol reagent (Invitrogen, Carlsbad, CA) and reverse-transcribed with qScript cDNA SuperMix (Quanta Biosciences, Gaithersburg, MD). qPCR was carried out using PerfeCTa SYBRGreen FastMix (Quanta Biosciences) on Roche Light Cycler 480 as previously described (Passeri et al., 2009). Comparative threshold (C_T_) values for the target genes were normalized with *ribosomal protein P0 (rpp0*) as a reference using 2^−CT(^*^target)^*/2^−CT(^*^rpp0)^*. Products of standard PCR were visualized by ethidium-bromide-stained 2% or 4% agarose gel electrophoresis. Primers are listed in supplementary material Table S1.

### Western blotting

Protein was extracted from a pool of ten dissected livers or from individual whole *Tg(l-fabp:Dbp-EGFP)* transgenic larvae, collected in RIPA buffer (20 mM Tris, pH 7.4, 150 mM NaCl, 2 mM EDTA, 1% NP-40, 10% glycerol) supplemented with protease inhibitors (Roche, Indianapolis, IN), mixed with 5× SDS loading buffer (250 mM Tris-Cl, 10% SDS, 0.5% bromophenol blue, 50% glycerol, 500 mM β-mercaptoethanol), heated for 10 minutes at 94°C and the entire extract was resolved by SDS-PAGE, transferred to a PVDF membrane (EMD Millipore Corporation, Billerica, MA) and incubated overnight with antibodies as indicated. Human hepatoma-derived VL-17A cells stably transfected with *ADH* and *CYP2E1* (Donohue et al., 2006) were used as a positive control (Howarth et al., 2012). Rabbit polyclonal anti-ADH1 (1:500, SC-22750, Santa Cruz Biotechnology); rabbit polyclonal anti-CYP2E1 (1:5000), a gift from Dr Jerome Lasker (Hackensack University Medical Center, Hackensack, NJ); mouse monoclonal anti-β-actin (1:5000, A5441, Sigma-Aldrich); anti-rabbit-HRP (1:5000, W401B, Promega); rabbit polyclonal anti-laminin (1:100, L9393, Sigma-Aldrich); anti-mouse-HRP (1:5000, 715-035-150, Jackson ImmunoResearch Laboratories); and anti-GFP (1:1000, Aves Labs) were used. Secondary antibodies from Molecular Probes (goat anti-chicken GFP, 1:200 and donkey anti-rabbit far red, 1:200) were used for immunofluorescence.

### Ethanol concentration assay

The Ethanol L3K^®^ Assay (Genzyme Diagnostics P.E.I. Inc., Charlottetown, Canada) was used according to the manufacturer’s instructions. In brief, larvae were collected in RIPA buffer at a volume of 2 μl/larva, homogenized by sonication, and pelleted by centrifugation. The supernatant was incubated in the Ethanol L3K^®^ Reagent at 1:50 ratio for 20 minutes. Absorbance at 340 nm was measured and normalized by subtracting the absorbance of untreated larvae. Ethanol concentration was calculated according to standard curve and a conversion factor of 3 was used assuming the volume of one larva was 1 μl {conversion factor=[volume of lysis buffer + (number of larvae×volume of one larva)]/(number of larvae×volume of one larva)}. Water collected from a dish immediately after larvae were transferred to fish water containing 350 mM ethanol at a density of 1 larva/ml was used as the ‘t=0 with larvae’ sample. In parallel, a dish with only 350 mM ethanol was used as the ‘t=0 without larvae’ sample. Plates were wrapped in paraffin and media collection was repeated at the end of the incubation. The water samples were incubated in the Ethanol L3K^®^ Reagent at 1:200 ratio.

### H_2_O_2_ measurements

5-(and-6)-chloromethyl-2′,7′-dichlorodihydrofluorescein diacetate, acetyl ester (CM-H_2_DCFDA; Invitrogen) was used to measure ROS. Larvae were treated with 5 μM CM-H_2_DCFDA for 90 minutes and fluorescence of the media was measured in duplicate at 485 nm excitation/538 nm emission wavelengths on a SpectroMax M5e Multi-Mode Microplate Reader (Molecular Devices, Sunnyvale, CA). Incubation solution without larvae was used as blank, and the arbitrary units of fluorescence in ethanol-exposed larvae were normalized to that of untreated fish.

### Imaging, data processing and statistics

Live *Tg(fabp10:dsRed)* larvae were treated and imaged as described (Howarth et al., 2011; Howarth et al., 2013). *Tg(l-fabp:Dbp-EGFP)* larvae were fixed overnight with 4% paraformaldehyde in 1× PBS. Between seven and ten larvae per treatment were imaged as described above. Images were taken using a Nikon SMZ1500 stereomicroscope with a Nikon Digital Sight camera. Images were minimally processed using Adobe Photoshop CS4 and Adobe Illustrator CS4, were inverted using Adobe Photoshop CS4, and the fluorescence intensity was quantified using ImageJ.

*Tg(hand2:EGFP)^pd24^* larvae were treated and processed as described (Yin et al., 2012) by imaging on a Zeiss Pascal confocal microscope, and image processing and cell counting were conducted using Fiji (http://fiji.sc/Fiji). Prism 5.0c was used to plot all graphs and conduct statistical analyses, including Fisher’s exact test, one-way ANOVA or Student’s *t*-test as appropriate.

Heatmaps of qPCR data were generated using GENE-E (Broad Institute, www.broadinstitute.org/cancer/software/GENE-E). Each square of the heatmap represents one data point for each gene and treatment. Colorization of the squares was determined via the median method in GENE-E. The median was calculated in the program for each row (gene) and subtracted from each data point. The resulting value was divided by the absolute deviation (AD) for the row. Data points were colored on a blue-white-red spectrum in which blue=3 ADs below the median or lower, white=median, and red=3 ADs above the median or higher.

## Supplementary Material

Supplementary Material
