# Supplementary material for: Ethanol metabolism and oxidative stress are required for unfolded protein response activation and steatosis in zebrafish with alcoholic liver disease
Source: Dis Model Mech. 2013 Jun 20;6(5):1213–26. doi: 10.1242/dmm.012195 (PMC3759341; doi:10.1242/dmm.012195)
Supplement: Supplementary Material [file supp_6_5_1213__index.html]

Supplementary Material 

# Ethanol metabolism and oxidative stress are required for unfolded protein response activation and steatosis in zebrafish with alcoholic liver disease

## DMM012195 Supplementary Material

**Files in this Data Supplement:**

- **Supplementary Material PDF**
