# Supplementary material for: Ethanol metabolism and oxidative stress are required for unfolded protein response activation and steatosis in zebrafish with alcoholic liver disease
Source: Dis Model Mech. 2013 Jun 20;6(5):1213–26. doi: 10.1242/dmm.012195 (PMC3759341; doi:10.1242/dmm.012195)
Supplement: Supplementary Material [file supp_012195_DMM012195.pdf]

Supplemental material

Supplemental Figure S1. Ethanol induces lordosis and edema in a dose- and time-dependent manner. (A) Zebrafish larvae were exposed to either 0 mM or 350 mM ethanol for 32 hours starting from 96 hpf to 128 hpf. Larvae were scored at 4-hour time intervals as affected if they displayed at least one abnormality (death, lordosis, edema, or a combination of phenotypes). The average of ten clutches is plotted (n=442 for each cohort, mean ± s.e.m.); statistical analysis was determined by two-way ANOVA and Bonferroni's post-hoc test. Except for the 4 hours, all time points on the 350 mM curve are significantly different ($p<0.001$) compared to 0 hours as well as corresponding time points on the 0 mM curve. The points marked with (a) are not statistically different from one another. **(B)** Larvae treated with 350 mM ethanol were scored for lordosis and edema over 32 hours of exposure. Note that most fish had both phenotypes. The average of thirteen clutches (n=562, mean ± s.e.m.) is plotted for each abnormality; statistical analysis of each phenotype was calculated by one-way ANOVA and Tukey's post-hoc test. All time points starting from 8 hours on the lordosis curve and from 12 hours on the edema curve are significantly different ($p<0.001$) compared to the time point of 0 hours. All time points on both curves are significantly different ($p<0.001$) from the time point of 32 hours except for 28 hours as indicated by (a) and (b) for lordosis and edema, respectively.

Supplemental Figure S2. Ethanol induces UPR. (A) Livers dissected from larvae exposed to 0 or 350 mM ethanol at 96-128 hpf were subject to standard PCR with *xbp1* primers, and the products were run in 4% agarose gel to achieve efficient separation of spliced and unspliced bands. The image is inverted. The spliced and unspliced bands were quantified, added to make 100%. Fold changes were calculated by normalizing the percentage of spliced bands of ethanol-treated samples to that of untreated samples and plotted in Figure 2B. **(B)** Immunoblot of Bip

and phosphorylated Eif2α of livers dissected from larvae exposed to 0 or 350 mM ethanol at 96-128 hpf. For each time point, protein was extracted from exactly 20 livers.

Supplemental Figure S3. Homologs of ethanol metabolizing enzymes are expressed in zebrafish. (A) The protein sequence alignment of zebrafish (*Danio rerio*; Dr) Cyp2y3 and Cyp2p6 with that of human CYP2E1 (Hs) and mouse Cyp2e1 (Mm). Cyp2y3 and Cyp2p6 show 43% identity to human CYP2E1. The pink box highlights the substrate-binding site, which is also a part of the antibody-binding site. **(B)** Representative PCR agarose gels of *adh5*, *adh8a*, *adh8b*, *cyp2y3*, *cyp2p6*, *aldh2* performed on cDNA prepared from a pool of dissected livers from control (–) and larvae treated with 350 mM ethanol (+) for 32 hours. *rpp0* serves as a reference gene. **(C-E)** Immunoblots of proteins from 10-25 livers per sample using antibodies raised against human ADH1 and CYP2E1. VL-17A cells stably expressing mouse Adh1 and human CYP2E1 served as a positive control.

Supplemental Figure S4. Optimization of CMZ, 4MP, CYA, and OAc in zebrafish larvae. Viability of larvae treated with 0-100 μM CMZ (**A**)**,** or 0-15 mM 4MP (**C**), or 0-10 mM CYA (**E**), or 0-500 mM OAc (**F**) was scored and plotted from the average of at least four clutches. The dashed lines in each plot indicate the optimal concentration. **(B)** Expression of CYP2E1-immunoreactive proteins is assessed by immunoblotting a pool of 10 zebrafish livers treated as shown. Because CMZ is a suicide inhibitor that results in the degradation of Cyp2e1 homolog, the dose of CMZ that minimizes the ethanol-induced Cyp2e1 expression would be the optimal concentration, which was determined to be 100 μM. **(D)** Western blotting of liver protein extracts using an antibody that was raised against human CYP2E1. Because 4MP is a competitive inhibitor of Cyp2e1, the lowest dose that stabilizes this enzyme in the absence of ethanol would be the optimal concentration of 4MP, which was determined as 1 mM.

Supplemental Figure S5. Optimization of H_2O_2 exposure doses to zebrafish larvae as assessed by survival and ROS production. **(A)** Zebrafish larvae were exposed to H_2O_2 at 0 mM, 0.4 mM (0.0010%), 0.9 mM (0.0020%), 1.0 mM (0.0023%), 1.3 mM (0.0030%), 1.7 mM (0.0040%), 2.0 mM (0.0047%), 2.1 mM (0.0050%), 2.6 mM (0.0060%), 3.0 mM (0.0070%), 3.4 mM (0.0080%), 3.8 mM (0.0090%), 4.0 mM (0.0094%), 4.3 mM (0.0100%) concentrations for 24 hours starting from 96 to 120 hpf. Viability at 24 hours was scored and plotted from the average of four clutches (n=60 larvae per treatment, mean ± s.e.m.); *** $p<0.001$ vs. 0 mM by one-way ANOVA and Tukey's post-hoc test. Note that 2.1 mM concentration is the lowest dose that introduces mortality. Interestingly, the survivors of any dose did not exhibit any phenotypic abnormality. **(B)** Generation of ROS by whole larvae treated as in **(A)** was measured by CM-H_2DCFDA fluorescence. Data in each concentration was normalized to 0 mM H_2O_2 and plotted from the average of four clutches. **(C)** Comparative survival study of zebrafish larvae exposed to 0 mM, 350 mM, 100 mM ethanol, 2.1 mM H_2O_2, and 100 mM ethanol in combination with 2.1 mM H_2O_2. Viability at 24 hours was scored and plotted from the average of three clutches. Note that the combination of low dose ethanol and H_2O_2 showed a synergistic response. **(D)** Comparative analysis of ROS production by whole larvae treated as in **(C)**. Data in each treatment was normalized to untreated larvae (0 mM EtOH + 0 mM H_2O_2) and plotted from the average of three clutches. The reason why the combination of low dose of ethanol and H_2O_2 did not increase ROS production may contribute to the fact that the ROS measurement was taken from the survivors only, which was about 50% of the initial population. It is interesting and scary to note that 350 mM ethanol that caused only 10% mortality (Figure 1B, 24 hours) produced high levels of ROS that is comparable to that of 3.8 mM (0.0090%) H_2O_2 exposure, which resulted in 95% mortality (Figure S5B).

Supplemental Figure S6. Ethanol-induced HSC activation is partially rescued by CMZ assessed by laminin deposition. (A-D) show confocal single plane images of *Tg(hand2:EGFP)* expression in the HSCs of larvae treated under different conditions from 96 hpf to 120 hpf. The larvae were fixed immediately after the treatment at 120 hpf. Arrows point to individual HSCs. In control and CMZ-treated larvae, HSCs send out long and multiple cellular processes. Moreover, these HSCs are well segregated from one another. In ethanol (EtOH)-treated larvae, HSCs lose the complex processes, become elongated, and tend to cluster together. In larvae treated with both CMZ and ethanol, the HSC phenotypes seem to be partially suppressed as these cells form short and multiple cellular processes (arrows in **D**). **(A'-D')** show the same images of **(A-D)**, but with laminin staining. Ethanol treatment triggers laminin deposition (arrows in **C'**), whereas co-treatment with ethanol and CMZ suppresses laminin deposition. The blue staining in A' and B' is due to autofluorescence of the red blood cells. **(A"-D")** show three-dimensional projection of the confocal Z-stack that scanning through the whole livers in **(A-D)**. In ethanol-treated liver, HSCs are clustered together, whereas in control, CMZ, and CMZ and ethanol co-treated liver, HSCs were well separated from one another. In **(A-D")** white dashed lines outline the periphery of the liver. Scale bar: 20 μm shown in **(A)**. **(E)** Quantification of whole mount oil red O staining in 120 hpf larvae that were either untreated (N/T), or pre-treated with 3 mM CYA, 40 mM OAc at 94 hpf and then exposed to 0 or 350 mM ethanol at 96 hpf for 24 hours. Note that neither of these treatments affected the ethanol-induced steatosis.

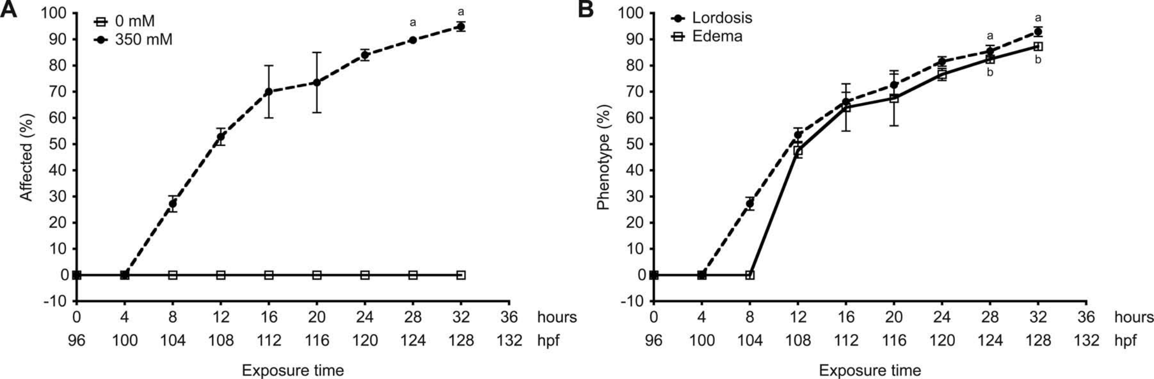
A
0 mM
350 mM
Affected (%)
a
a
100
90
80
70
60
50
40
30
20
10
0
-10
0
4
8
12
16
20
24
28
32
36
hours
96
100
104
108
112
116
120
124
128
132
hpf
Exposure time
B
Lordosis
Edema
Phenotype (%)
a
a
b
b
Exposure time

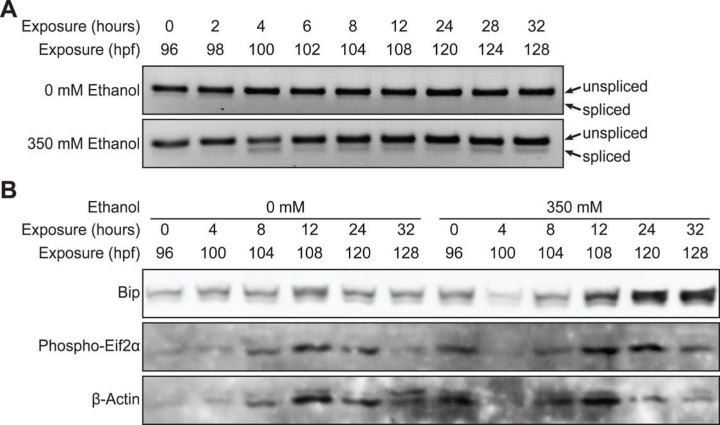
A
Exposure (hours) 0 2 4 6 8 12 24 28 32
Exposure (hpf) 96 98 100 102 104 108 120 124 128
0 mM Ethanol
unspliced
spliced
350 mM Ethanol
unspliced
spliced
B
Ethanol
0 mM
350 mM
Exposure (hours) 0 4 8 12 24 32 0 4 8 12 24 32
Exposure (hpf) 96 100 104 108 120 128 96 100 104 108 120 128
Bip
Phospho-Eif2α
β-Actin

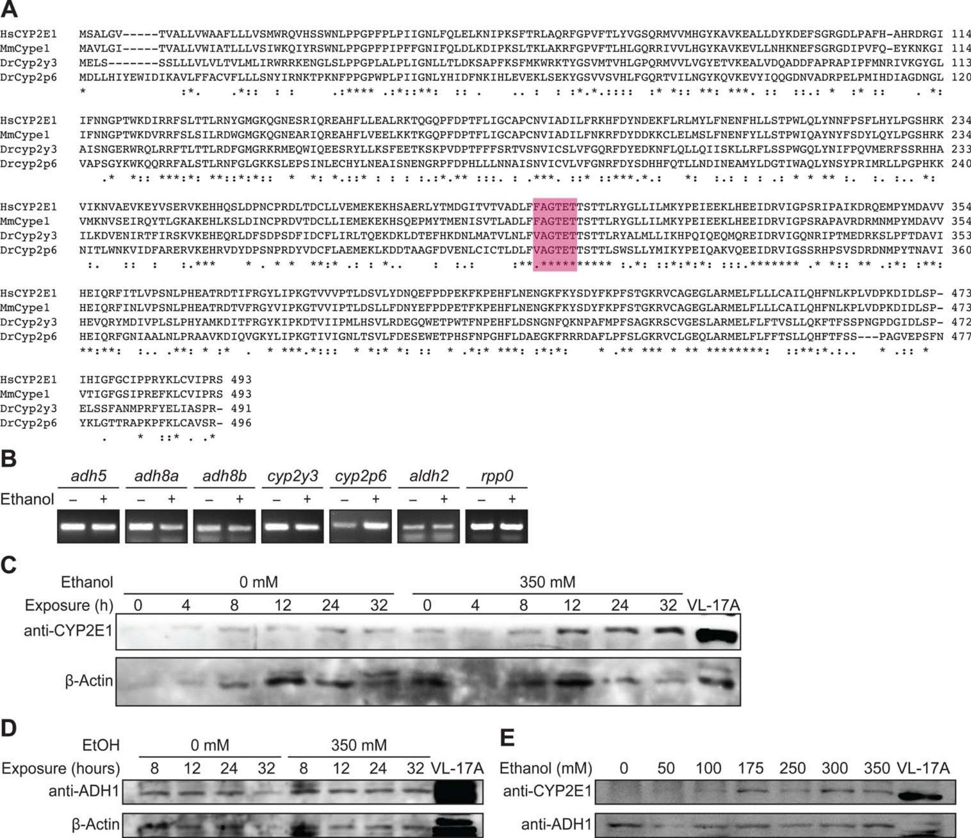
A
HsCYP2E1 MSALGV-----TVALLVWAAFLLLVSMWRQVHSSWNLPPGPFPLPIIGNLFQLELKNIPKSFTRLAQRFGPVFTLYVGSQRMVVMHGYKAVKEALLDYKDEFSGRGDLPAFH-AHRDRGI 114
MmCype1 MAVLGI-----TVALLVWIATLLLVSIWKQIYRSWNLPPGPFPIPFFGNIFQLDLKDIPKSLTKLAKRFGPVFTLHLGQRRIVVLHGYKAVKEVLLNHKNEFSGRGDIPVFQ-EYKNKGI 114
DrCyp2y3 MELS-------SSLLLVLVLTVLMLIRWRRKENGLSLPPGPLALPLIGNLLTLDKSAPFKSFMKWRKTYGSVMTVHLGPQRMVVLVGYETVKEALVDQADDFAPRAPIPFMNRIVKGYGL 113
DrCyp2p6 MDLLHIYEWIDIKAVLFFACVFLLLSNYIRNKTPKNFPPGPWPLPIIGNLYHIDFNKIHLEVEKLSEKYGSVVSVHLFGQRTVILNGYKQVKEVYIQQGDNVADRPELPMIHDIAGDNGL 120
HsCYP2E1 IFNNGPTWKDIRRFSLTTLRNYGMGKQGNESRIQREAHFLLEALRKTQGQPFDPTFLIGCAPCNVIADILFRKHFDYNDEKFLRLMYLFNENFHLLSTPWLQLYNNFPSFLHYLPGSHRK 234
MmCype1 IFNNGPTWKDVRRFSLSILRDWGMGKQGNEARIQREAHFLVEELKKTKGQPFDPTFLIGCAPCNVIADILFNKRFDYDDKKCLELMSLFNENFYLLSTPWIQAYNYFSDYLQYLPGSHRK 234
Drcyp2y3 AISNGERWRQLRRFTLTTLRDFGMGRKRMEQWIQEESRYLLKSFEETKSKPVDPTFFFSRTVSNVICSLVFGQRFDYEDKNFLQLLQIISKLLRFLSSPWGQLYNIFPQVMERFSSRHHA 233
Drcyp2p6 VAPSGYKWKQQRRFALSTLRNFGLGKKSLEPSINLECHYLNEAISNENGRPFDPHLLLNNAISNVICVLVFGNRFDYSDHHFQTLLNDINEAMYLDGTIWAQLYNSYPRIMRLLPGPHKK 240
HsCYP2E1 VIKNVAEVKEYVSERVKEHHQSLDPNCPRDLTDCLLVEMEKEKHSAERLYTMDGITVTVADLFFAGTETTSTTLRYGLLILMKYPEIEEKLHEEIDRVIGPSRIPAIKDRQEMPYMDAVV 354
MmCype1 VMKNVSEIRQYTLGKAKEHLKSLDINCPRDVTDCLLIEMEKEKHSQEPMYTMENISVTLADLFFAGTETTSTTLRYGLLILMKYPEIEEKLHEEIDRVIGPSRAPAVRDRMNMPYMDAVV 354
DrCyp2y3 ILKDVENIRTFIRSKVKEHEQRLDFSDPSDFIDCFLIRLTQEKDKLDTEFHKDNLMATVLNLFVAGTETTSTTLRYALMLLIKHPQIQEQMQREIDRVIGQNRIPTMEDRKSLPFTDAVI 353
DrCyp2p6 NITLWNKVIDFARERVKEHRVDYDDPSNPRDYVDCFLAEMEKLKDDTAAGFDVENLCICTLDLFVAGTETTSTTLSWSLLYMIKYPEIQAKVQEEIDRVIGSSRHPSVSDRDNMPYTNAVI 360
HsCYP2E1 HEIQRFITLVPSNLPHEATRDTIFRGYLIPKGTVVVPTLDSVLYDNQEFPDPEKFKPEHFLNENGKFKYSDYFKPFSTGKRVCAGEGLARMELFLLLCAILQHFNLKPLVDPKDIDLSP- 473
MmCype1 HEIQRFINLVPSNLPHEATRDTVFRGYVIPKGTVVIPTLDSLLFDNYEFPDPETFKPEHFLNENGKFKYSDYFKPFSTGKRVCAGEGLARMELFLLLCAILQHFNLKPLVDPKDIDLSP- 473
DrCyp2y3 HEVQRYMDIVPLSLPHYAMKDITFRGYKIPKDTVIIPMLHSVLRDEGQWETPWTFNPEHFLDSNGNFQKNPAFMPFSAGKRSCVGESLARMELFLFTVSLLQKFTFSSPNGPDGIDLSP- 472
DrCyp2p6 HEIQRFGNIAALNLPRAAVKDIQVGKYLIPKGTIVIGNLTSVLFDESEWETPHSFNPGHFLDAEGKFRRRDAFLPFSLGKRVCLGEQLARMELFLFFTSLLQHFTFSS---PAGVEPSFN 477
HsCYP2E1 IHIGFGCIPPRYKLCVIPRS 493
MmCype1 VTIGFGSIPREFKLCVIPRS 493
DrCyp2y3 ELSSFANMPRFYELIASPR- 491
DrCyp2p6 YKLGTTRAPKPFKLCAVSR- 496
B
adh5
adh8a
adh8b
cyp2y3
cyp2p6
aldh2
rpp0
Ethanol – + – + – + – + – + – + – +
C
Ethanol 0 mM 350 mM
Exposure (h) 0 4 8 12 24 32 0 4 8 12 24 32 VL-17A
anti-CYP2E1
β-Actin
D
EtOH 0 mM 350 mM
Exposure (hours) 8 12 24 32 8 12 24 32 VL-17A
anti-ADH1
β-Actin
E
Ethanol (mM) 0 50 100 175 250 300 350 VL-17A
anti-CYP2E1
anti-ADH1

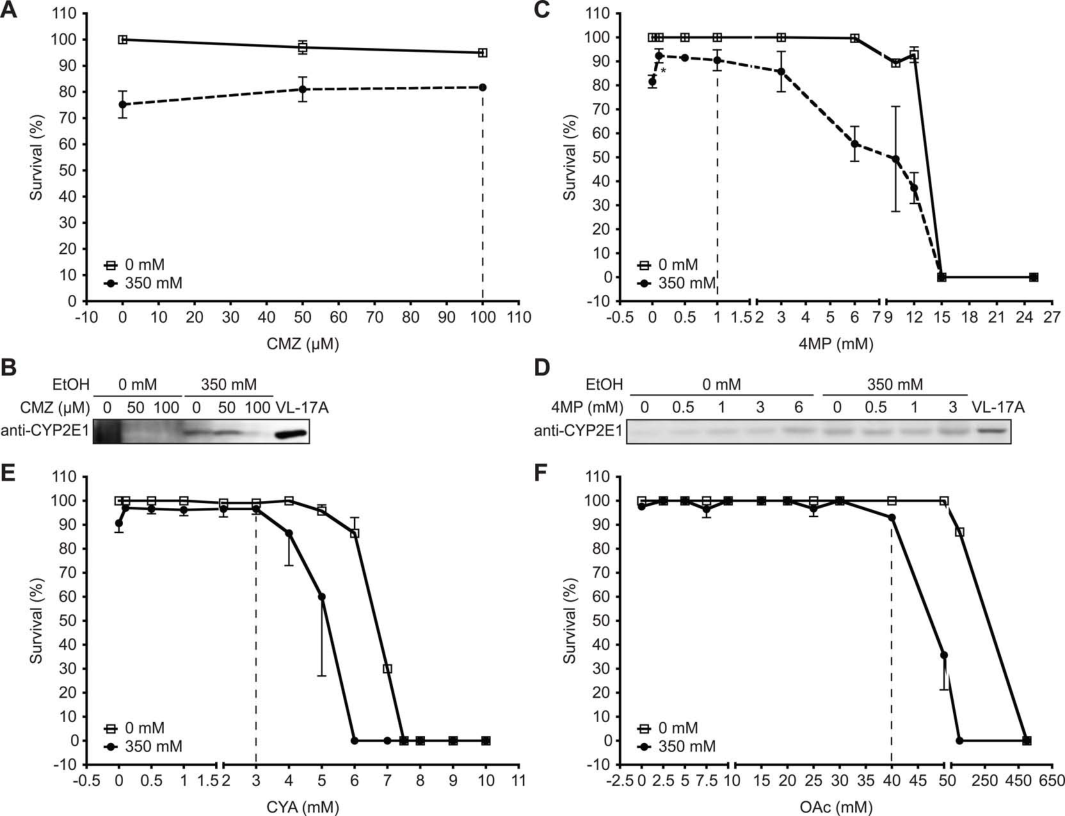

A
Survival (%)
CMZ (μM)
0 mM
350 mM
B
EtOH
0 mM
350 mM
CMZ (μM)
0 50 100 0 50 100 VL-17A
anti-CYP2E1
C
Survival (%)
4MP (mM)
0 mM
350 mM
D
EtOH
0 mM
350 mM
4MP (mM)
0 0.5 1 3 6 0 0.5 1 3 VL-17A
anti-CYP2E1
E
Survival (%)
CYA (mM)
0 mM
350 mM
F
Survival (%)
OAc (mM)
0 mM
350 mM

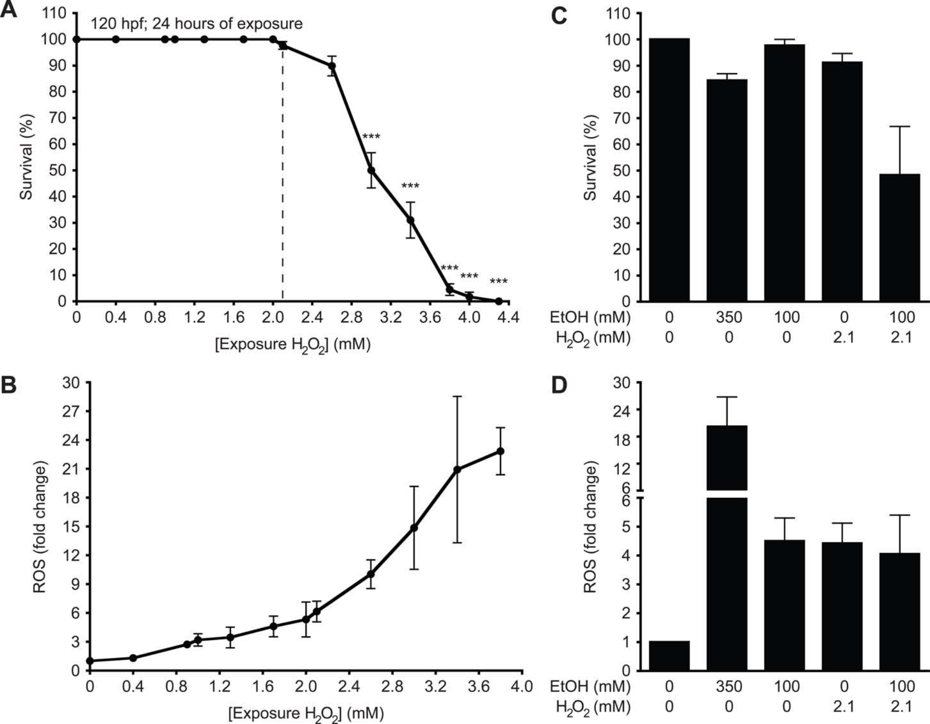
A
120 hpf; 24 hours of exposure
Survival (%)
[Exposure H_2O_2] (mM)
***
B
ROS (fold change)
[Exposure H_2O_2] (mM)
C
Survival (%)
EtOH (mM) 0 350 100 0 100
H_2O_2 (mM) 0 0 0 2.1 2.1
D
ROS (fold change)
EtOH (mM) 0 350 100 0 100
H_2O_2 (mM) 0 0 0 2.1 2.1

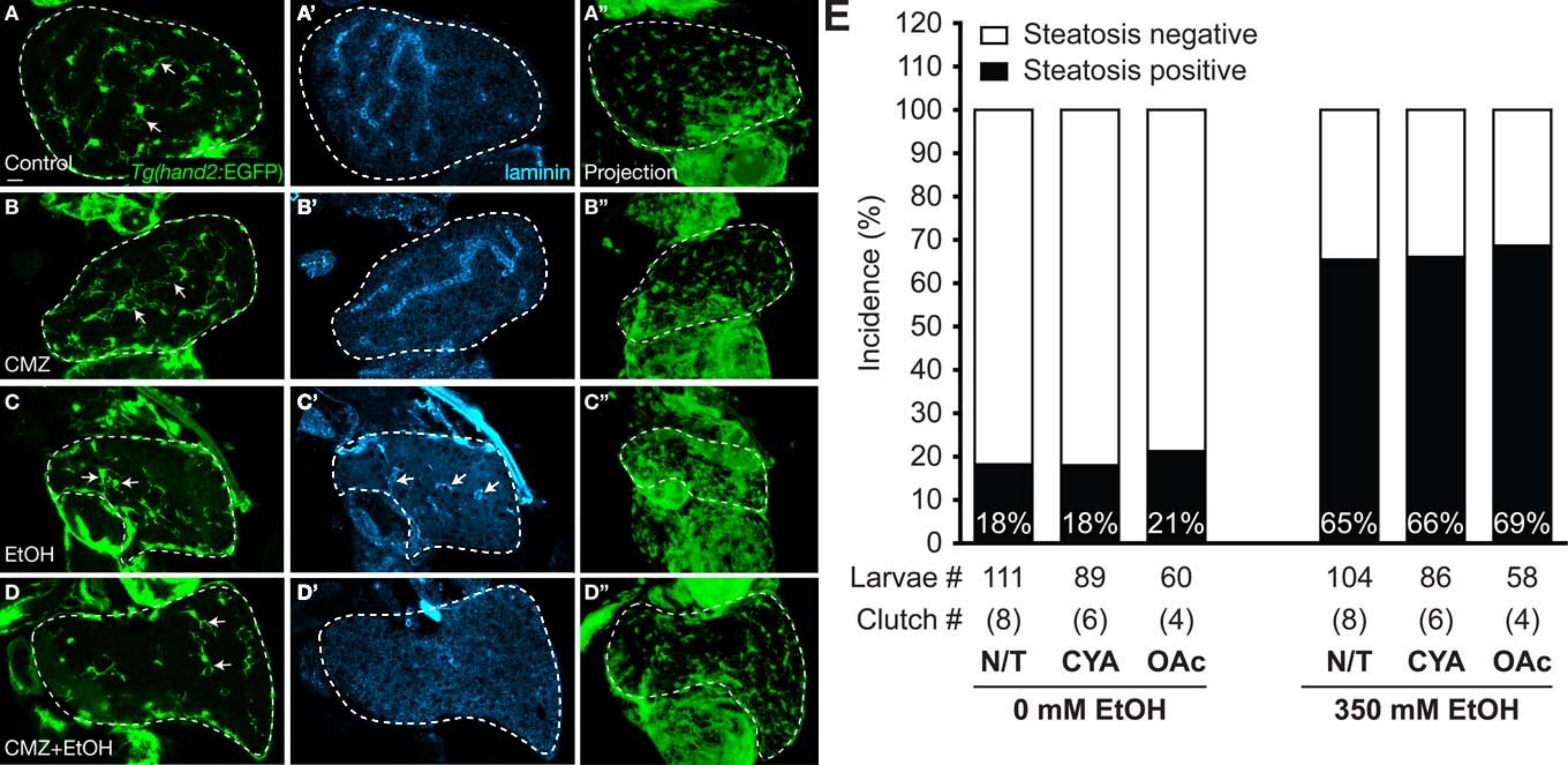

A
A'
A''
Control
Tg(hand2:EGFP)
laminin
Projection
B
B'
B''
CMZ
C
C'
C''
EtOH
D
D'
D''
CMZ+EtOH
E
Steatosis negative
Steatosis positive
Incidence (%)
120
110
100
90
80
70
60
50
40
30
20
10
0
18%
18%
21%
65%
66%
69%
Larvae # 111 89 60 104 86 58
Clutch # (8) (6) (4) (8) (6) (4)
N/T CYA OAc N/T CYA OAc
0 mM EtOH
350 mM EtOH

Table S1. Primer sequences used for conventional and quantitative, real-time PCR (qPCR)

Gene name	Forward	Reverse
a1at	CATGTTGGGTCACAGTCAGG	CGATTTCAGGCTTGGAGAA
acc1	GCAAGTGTGGTTCCCTGATT	TCATGAAGGTCAGCGAACTG
adh5	CCTTGAATTGAAGCCTGAGC	CAACTGAAACCCGACACACA
adh8a	AGCTTTCTCTGCAGCTGTTTG	GCCAAGGATTACTTTTTCTTGC
adh8b	ACAGATGGAAATTGAGACATTCC	TTTCAACACGTGACAAGATCG
bip	ATCAGATCTGGCCAAAATGC	CCACGTATGACGGAGTGATG
cat	CGCTTCTGTTTCCGTCTTTC	GGAATCCCTCGATCACTGAA
cnx	CATCTCTGCCCTCCTACTGC	TCCACAAACTCCTCCAGGTC
cyp2p6	ACTGAGACCACCTCCACCAC	CATTGGTGTAGGGCATGTTG
cyp2y3	TATTCCCATGCTGCACTCTG	AGGAGCGTTTACCTGCAGAA
derl1	TTTCGACTTGGTGACGACTG	CAGAGCCCTCGTCACTTTTC
dnajc3	TCCCATGGATCCTGAGAGTC	CTCCTGTGTGTGAGGGGTCT
edem1	GACAGCAGAAACCCTCAAGC	CATGGCCCTCATCTTGACTT
fasn	GAGAAAGCTTGCCAAACAGG	GAGGGTCTTGCAGGAGACAG
gclc	AACCGACACCCAAAGATTCAGCACT	CCATCATCCTCTGGAAACACCTCC
gpx1a	GTAAACCAGCGGCTTCTACG	GGCACTTTAATCATGACTGCAC
gr	ATTGGCAGAGAACCCAACAC	ACATCCCCGACTGCATAGAC
grp94	AGCAAGACCGAGACCGTAGA	CTCCCAATCCCACACAGTCT
hmgcra	CTGAGGCTCTGGTGGACGTG	CGCCGCAGCTACGATGTTGGCG
hmgcs1	CTCACTCGTGTGGACGAGAA	GATACGGGGCATCTTCTTGA
mvk	CACACTTTTGCGACCAGAGA	CAGCAGCAATGAGCATCTGT
pdi	GATTCGGTGCCATCAGAGAT	TCCAGTCACAGCGACCACTA
prdx1	CGCGAGTTCACTTTCATTCA	TGACAAACGGACATCTCCAA
rpp0	CTGAACATCTCGCCCTTCTC	TAGCCGATCTGCAGACACAC
saa	CGTGCCTACCAGCATATGAA	CAGCATCTGAATTGCCTCTG
scd	GGTGTTGATGTGCTTCGTTG	CCGATGTTTCCATCATAGGG
sod2	AGCGTGACTTTGGCTCATTT	ATGAGACCTGTGGTCCCTTG
srebp1	ACTCTTCTGGTGTGGCTGCT	GAGCCTTCAGACACGTCCTC
srebp2	CACTCACACAAGCACACACG	ACCTGGTTCTGGATGAATCG
txnl1	TCACAATGGCCTTCAATCAA	CCAGACTCTGTGTGGCTTCA
txnl4a	CACAATGGCTGGCAAGTAGA	CTGGCACTTCTGTGATGTCC

Table S2. dCt values of qPCR results in Figure 4C

Treatment	Replicate	*gr*	*txnl1*	*txnl4*	*gpx1*	*sod2*	*a1at*	*saa*
0 mM EtOH	1	0.00210	0.00832	0.00149	0.1350	0.0141	2.537	0.0000195
	2	0.00207	0.00714	0.00138	0.1480	0.0149	2.514	0.0000188
	3	0.00288	0.00753	0.00125	0.1980	0.0191	2.502	0.0000193
	4	0.00256	0.00873	0.00116	0.2040	0.0199	2.848	0.0000239
	5	0.00174	0.00510	0.00097	0.0940	0.0213	2.537	0.0000026
	6	0.00461	0.00960	0.00119	0.1470	0.0282	2.770	0.0000380
	**Median**	**0.00233**	**0.00793**	**0.00122**	**0.1475**	**0.0195**	**2.537**	**0.0000194**
350 mM EtOH	1	0.01581	0.04737	0.00600	0.3060	0.0298	7.413	0.0001859
	2	0.01506	0.04682	0.00638	0.2770	0.0296	7.013	0.0001271
	3	0.01475	0.04230	0.00592	0.4530	0.0372	5.816	0.0001645
	4	0.01690	0.05982	0.00677	0.5030	0.0342	6.558	0.0001618
	5	0.01686	0.04533	0.00517	0.2270	0.0354	6.758	0.0000520
	6	0.01625	0.04259	0.00456	0.3760	0.0364	6.869	0.0000457
	**Median**	**0.01603**	**0.04608**	**0.00596**	**0.3410**	**0.0348**	**6.814**	**0.0001445**
CMZ + EtOH	1	0.01123	0.02210	0.00576	0.1950	0.0190	5.327	0.0000443
	2	0.01007	0.01795	0.00545	0.1770	0.0160	5.266	0.0000362
	3	0.01043	0.01833	0.00606	0.1810	0.0198	4.084	0.0000374
	4	0.01033	0.02492	0.00589	0.1820	0.0201	4.879	0.0000213
	5	0.04759	0.03139	0.00414	0.4160	0.0266	5.683	0.0000108
	6	0.04859	0.03349	0.00506	0.3060	0.0271	6.049	0.0000057
	**Median**	**0.01083**	**0.02351**	**0.00561**	**0.1885**	**0.0200**	**5.297**	**0.0000288**
4MP + EtOH	1	0.02936	0.02498	0.00552	0.2730	0.0276	4.248	0.0000287
	2	0.03380	0.02727	0.00663	0.2590	0.0320	4.367	0.0000325
	3	0.03691	0.02604	0.00596	0.3410	0.0305	4.428	0.0000416
	4	0.03303	0.03147	0.00556	0.3220	0.0260	4.902	0.0000235
	5	0.01667	0.02001	0.00600	0.2470	0.0236	5.110	0.0000171
	6	0.02071	0.02671	0.00774	0.2710	0.0241	5.040	0.0000042
	**Median**	**0.03120**	**0.02638**	**0.00598**	**0.2720**	**0.0268**	**4.665**	**0.0000261**
AA + EtOH	1	0.01411	0.02330	0.00501	0.2040	0.0210	5.723	0.0000520
	2	0.01434	0.00238	0.00507	0.1930	0.0238	5.696	0.0000264
	3	0.01311	0.02200	0.00476	0.2300	0.0203	3.864	0.0000921
	4	0.01434	0.02977	0.00491	0.2120	0.0171	3.724	0.0001060
	5	0.01924	0.02396	0.00386	0.2630	0.0214	5.005	0.0000106
	6	0.01982	0.03665	0.00411	0.2140	0.0242	6.394	0.0000088
	**Median**	**0.01434**	**0.02363**	**0.00484**	**0.2130**	**0.0212**	**5.351**	**0.0000392**
NAC + EtOH	1	0.01746	0.01858	0.00513	0.2100	0.0223	4.387	0.0000190
	2	0.01795	0.02390	0.00551	0.2410	0.0217	4.936	0.0000398
	3	0.01659	0.02784	0.00603	0.2080	0.0247	4.735	0.0000564
	4	0.01482	0.02876	0.00592	0.0020	0.0231	4.713	0.0000711
	5	0.01485	0.03265	0.00345	0.2200	0.0185	4.387	0.0000154
	6	0.01942	0.02896	0.00347	0.2630	0.0218	4.428	0.0000533
	**Median**	**0.01703**	**0.02830**	**0.00532**	**0.2150**	**0.0221**	**4.571**	**0.0000466**

Table S3. dCt values of qPCR results in Figure 5A

Treatment	Replicate	*bip*	*dnajc3*	*grp94*	*edem1*	*cnx*	*pdi*
**0 mM EtOH**	**1**	0.0041	0.0016	0.0034	0.0037	0.0154	0.0819
	**2**	0.0039	0.0019	0.0039	0.0039	0.0170	0.0894
	**3**	0.0044	0.0018	0.0040	0.0032	0.0212	0.1058
	**4**	0.0047	0.0018	0.0040	0.0036	0.0223	0.1096
	**5**	0.0072	0.0018	0.0034	0.0035	0.0158	0.0726
	**6**	0.0059	0.0030	0.0060	0.0059	0.0250	0.0958
	**Median**	**0.00455**	**0.00180**	**0.00395**	**0.0037**	**0.0191**	**0.0926**
**350 mM EtOH**	**1**	0.2437	0.0261	0.1419	0.01392	0.1497	0.2186
	**2**	0.2512	0.0262	0.1381	0.01317	0.1381	0.176
	**3**	0.1601	0.0239	0.1098	0.01495	0.1365	0.198
	**4**	0.1998	0.0254	0.1049	0.01636	0.1282	0.2171
	**5**	0.1731	0.0308	0.0896	0.01152	0.1207	0.1843
	**6**	0.1708	0.0275	0.1256	0.01326	0.1227	0.2146
	**Median**	**0.18645**	**0.02615**	**0.11770**	**0.0136**	**0.1324**	**0.2063**
**CMZ + EtOH**	**1**	0.1692	0.0249	0.0992	0.01257	0.118	0.1843
	**2**	0.1553	0.0239	0.002	0.01243	0.0963	0.1731
	**3**	0.1571	0.0236	0.0907	0.01146	0.1196	0.1788
	**4**	0.1568	0.025	0.0804	0.01209	0.1219	0.1539
	**5**	0.0902	0.0145	0.0423	0.01139	0.1145	0.1939
	**6**	0.1413	0.016	0.0742	0.0132	0.1108	0.1882
	**Median**	**0.15605**	**0.02375**	**0.07730**	**0.0123**	**0.1163**	**0.1816**
**4MP + EtOH**	**1**	0.13000	0.01510	0.06180	0.01125	0.10980	0.13460
	**2**	0.13490	0.01720	0.07250	0.01332	0.12190	0.15000
	**3**	0.12560	0.01740	0.07160	0.01206	0.12670	0.16690
	**4**	0.13210	0.01690	0.07020	0.01252	0.11030	0.17840
	**5**	0.13240	0.01910	0.06350	0.01335	0.14290	0.19480
	**6**	0.10410	0.01580	0.05790	0.01357	0.12970	0.15710
	**Median**	**0.13105**	**0.01705**	**0.06685**	**0.0129**	**0.1243**	**0.1620**
**AA + EtOH**	**1**	0.14100	0.02100	0.07750	0.01100	0.09430	0.20310
	**2**	0.11850	0.02350	0.07450	0.01120	0.10340	0.18510
	**3**	0.05160	0.01510	0.03920	0.00931	0.08320	0.14000
	**4**	0.05430	0.01590	0.04090	0.00749	0.08480	0.12790
	**5**	0.08840	0.01410	0.03400	0.00933	0.10610	0.18220
	**6**	0.10270	0.01590	0.05280	0.01115	0.10370	0.20450
	**Median**	**0.09555**	**0.01590**	**0.04685**	**0.0102**	**0.0989**	**0.1837**
**NAC + EtOH**	**1**	0.16880	0.02150	0.10580	0.01107	0.12470	0.17470
	**2**	0.19430	0.02530	0.12560	0.01281	0.11290	0.20540
	**3**	0.11850	0.01940	0.08040	0.01125	0.11320	0.18640
	**4**	0.11770	0.02040	0.06950	0.01168	0.11400	0.17470
	**5**	0.03550	0.00910	0.01850	0.00736	0.08780	0.13270
	**6**	0.02500	0.01120	0.02230	0.00973	0.09360	0.19030
	**Median**	**0.11810**	**0.01990**	**0.07495**	**0.0112**	**0.1131**	**0.1806**

Table S4. dCt values of qPCR results in Figure 5D

Treatment	Replicate	*bip*	*dnajc3*	*grp94*	*edem1*	*cnx*
**0 mM EtOH**	**1**	0.002613	0.001025	0.002947	0.003408	0.013018
	**2**	0.002913	0.001030	0.003401	0.003738	0.015303
	**3**	0.003988	0.001205	0.002583	0.002743	0.010821
	**4**	0.002879	0.000905	0.002218	0.003037	0.007903
	**5**	0.005238	0.001590	0.003440	0.003645	0.015553
	**6**	0.002438	0.001111	0.002371	0.002285	0.010525
	**Median**	**0.002896**	**0.001071**	**0.002765**	**0.003223**	**0.011920**
**100 mM EtOH**	**1**	0.005002	0.001328	0.005202	0.003424	0.016326
	**2**	0.004743	0.001337	0.004809	0.003704	0.017257
	**3**	0.003670	0.001583	0.004044	0.003817	0.012007
	**4**	0.003180	0.001194	0.003738	0.002461	0.009142
	**5**	0.003308	0.001793	0.003870	0.003970	0.012402
	**6**	0.005025	0.002988	0.006331	0.004539	0.012809
	**Median**	**0.004206**	**0.001460**	**0.004427**	**0.003761**	**0.012605**
**2.1 mM H_2O_2**	**1**	0.038119	0.007025	0.057114	0.006770	0.047366
	**2**	0.041426	0.006073	0.054284	0.006031	0.051237
	**3**	0.186856	0.007512	0.105355	0.005786	0.043788
	**4**	0.141610	0.010237	0.118257	0.006928	0.046930
	**5**	0.062789	0.008790	0.049721	0.004865	0.032804
	**6**	0.034118	0.004016	0.022718	0.004245	0.025208
	**Median**	**0.052108**	**0.007268**	**0.055699**	**0.005908**	**0.045359**
**H_2O_2 + EtOH**	**1**	0.079844	0.010120	0.067921	0.009530	0.058449
	**2**	0.085773	0.010453	0.075887	0.010356	0.077482
	**3**	0.092569	0.006003	0.042296	0.006101	0.045123
	**4**	0.103426	0.007256	0.073642	0.003799	0.039922
	**5**	0.300756	0.007634	0.089415	0.003852	0.050649
	**6**	0.102238	0.013508	0.086170	0.007357	0.052922
	**Median**	**0.097403**	**0.008877**	**0.074764**	**0.006729**	**0.051785**

Table S5. dCt values of qPCR results in Figure 5E

Treatment	Replicate	*gr*	*txnl1*	*txnl4*	*gpx1*	*sod2*	*cat*	*gclc*	*prdx1*
0 mM EtOH	1	0.002088	0.007885	0.001456	0.192109	0.017257	0.075016	0.003108	0.0000314
	2	0.001612	0.006723	0.001689	0.186425	0.017020	0.078201	0.003173	0.0000089
	3	0.001265	0.010027	0.001197	0.148651	0.015198	0.069348	0.004710	0.0000043
	4	0.001878	0.008649	0.001337	0.185137	0.015058	0.069992	0.005386	0.0000072
	5	0.001673	0.012316	0.002589	0.177595	0.020857	0.083427	0.005373	0.0000272
	6	0.004007	0.009775	0.001047	0.163421	0.014017	0.053909	0.004843	0.0000205
	**Median**	**0.001775**	**0.009212**	**0.001397**	**0.181366**	**0.016109**	**0.072504**	**0.004776**	**0.0000147**
100 mM EtOH	1	0.006880	0.007529	0.001869	0.263645	0.019641	0.086170	0.005119	0.0000523
	2	0.006785	0.006615	0.002208	0.253490	0.020428	0.082279	0.005563	0.0000430
	3	0.007616	0.010027	0.001494	0.190782	0.011733	0.054788	0.005448	0.0000226
	4	0.008354	0.010623	0.001325	0.207809	0.014147	0.070316	0.006976	0.0000541
	5	0.006479	0.011571	0.002461	0.205423	0.020240	0.091717	0.006073	0.0000929
	6	0.008201	0.010004	0.001368	0.168404	0.016289	0.057247	0.005826	0.0000206
	**Median**	**0.007248**	**0.010015**	**0.001682**	**0.206616**	**0.017965**	**0.076298**	**0.005694**	**0.0000476**
2.1 mM H_2O_2	1	0.003058	0.013292	0.002650	0.258816	0.032728	0.073302	0.006215	0.0000472
	2	0.003756	0.012721	0.002559	0.246558	0.029839	0.066986	0.005772	0.0000367
	3	0.003424	0.019148	0.001035	0.127332	0.022302	0.017020	0.003888	0.0000724
	4	0.007940	0.019550	0.002393	0.269184	0.046284	0.056851	0.004550	0.0000680
	5	0.004843	0.013109	0.001627	0.221186	0.027457	0.035239	0.005934	0.0000581
	6	0.003897	0.011177	0.002339	0.248847	0.015446	0.058449	0.006230	0.0000216
	**Median**	**0.003827**	**0.013200**	**0.002366**	**0.247703**	**0.028648**	**0.057650**	**0.005853**	**0.0000527**
H_2O_2 + EtOH	1	0.007652	0.020193	0.002093	0.257623	0.034118	0.028623	0.005373	0.0001641
	2	0.008451	0.024014	0.002781	0.302149	0.045753	0.032502	0.007812	0.0001988
	3	0.006585	0.011491	0.001477	0.201660	0.026645	0.031540	0.006646	0.0000843
	4	0.005962	0.019060	0.001579	0.130912	0.027712	0.012988	0.005263	0.0000879
	5	0.008912	0.025091	0.002218	0.184710	0.029839	0.013792	0.010574	0.0001637
	6	0.008953	0.022406	0.003464	0.226880	0.031395	0.041714	0.005588	0.0000978
	**Median**	**0.008051**	**0.021299**	**0.002156**	**0.214270**	**0.030617**	**0.030082**	**0.006117**	**0.0001307**
